# Coccolithoviruses: A Review of Cross-Kingdom Genomic Thievery and Metabolic Thuggery

**DOI:** 10.3390/v9030052

**Published:** 2017-03-18

**Authors:** Jozef I. Nissimov, António Pagarete, Fangrui Ma, Sean Cody, David D. Dunigan, Susan A. Kimmance, Michael J. Allen

**Affiliations:** 1 Plymouth Marine Laboratory, Prospect Place, The Hoe, Plymouth PL1 3DH, UK; jnissimov@marine.rutgers.edu (J.I.N.); sukim@pml.ac.uk (S.A.K.); 2 Department of Marine and Coastal Sciences, Rutgers University, New Brunswick, NJ 08901, USA; 3 Department of Biology, University of Bergen, Bergen 7803, Norway; antonio.pagarete@uib.no; 4 Nebraska Center for Virology, University of Nebraska, Lincoln, NE 68583, USA; fma2@unl.edu (F.M.); wfr1995@gmail.com (S.C.); ddunigan2@unl.edu (D.D.D.)

**Keywords:** *E. huxleyi*, coccolithovirus, genome comparison, horizontal gene transfer, domains of life

## Abstract

Coccolithoviruses (*Phycodnaviridae*) infect and lyse the most ubiquitous and successful coccolithophorid in modern oceans, *Emiliania huxleyi*. So far, the genomes of 13 of these giant lytic viruses (i.e., *Emiliania huxleyi* viruses—EhVs) have been sequenced, assembled, and annotated. Here, we performed an in-depth comparison of their genomes to try and contextualize the ecological and evolutionary traits of these viruses. The genomes of these EhVs have from 444 to 548 coding sequences (CDSs). Presence/absence analysis of CDSs identified putative genes with particular ecological significance, namely sialidase, phosphate permease, and sphingolipid biosynthesis. The viruses clustered into distinct clades, based on their DNA polymerase gene as well as full genome comparisons. We discuss the use of such clustering and suggest that a gene-by-gene investigation approach may be more useful when the goal is to reveal differences related to functionally important genes. A multi domain “Best BLAST hit” analysis revealed that 84% of the EhV genes have closer similarities to the domain Eukarya. However, 16% of the EhV CDSs were very similar to bacterial genes, contributing to the idea that a significant portion of the gene flow in the planktonic world inter-crosses the domains of life.

## 1. Introduction

It has been more than 10 years since the complete genome sequencing of a giant double-stranded DNA-containing virus infecting the ubiquitous bloom-forming coccolithophorid species *Emiliania huxleyi* [1]. *Emiliania huxleyi* virus strain number 86 (EhV-86) was isolated in 1999 from the English Channel, and a subsequent analysis of its major capsid protein (MCP) and DNA polymerase genes placed it in a new separate genus, *Coccolithovirus*, within the family *Phycodnaviridae* [2,3,4]. The *Phycodnaviridae* comprises other giant viruses that infect algae, such as *Chlorella* sp. (e.g., PBCV-1), *Ectocarpus siliculousus* (e.g., EsV-1), *Micromonas pusilla* (e.g., MpV-SP1), *Chrysochromulina brevifilum* (e.g., CbV-PW1), and *Heterosigma akashiwo* (e.g., HaV01) [5,6]. Because of *E. huxleyi*’s global impact on biogeochemical cycles, the study of this host-virus system is particularly relevant. Interactions between *E. huxleyi* and EhVs have been investigated both in vivo, through large-scale semi-natural mesocosm experiments and natural *E. huxleyi* blooms [7,8,9,10,11], and in vitro [12,13,14,15,16], in laboratory experiments designed to elucidate specific aspects of the infection cycle, the infection dynamics and the host cellular response to infection [17].

As a result of these studies, considerable insight has been gained regarding (1) the EhV intein [18] and the expression profile of EhV genes during infection [19,20,21]; (2) the EhV life cycle, including its utilization of lipid rafts for budding from the host cells [22,23]; (3) host cellular processes in response to infection such as autophagy and the induction of programmed cell death (PCD) pathways [17,24,25,26,27]; (4) the manipulation of fatty acid and lipid metabolism within infected cells, leading to the production of virus-induced lipids crucial for the progression of the infection [10,28,29,30,31]; (5) vector transmission of EhVs in the natural environment via aerosols and zooplankton faecal pellets [32,33]; and (6) the co-occurring diversity of EhVs and their viruses in a range of habitats, including the Atlantic Ocean, Norwegian Fjords and coastal regions of the Black Sea [8,34,35,36,37,38]. Perhaps the most astonishing finding to date was the identification of a de novo sphingolipid biosynthesis pathway in the EhV genome [1,9,39], encoding virus-derived glycosphingolipids (vGSLs) that are crucial regulators of infection [28]. Thus, important parts of the metabolic activity in the planktonic realm (in this case lipid production) are viral-driven [40]. It has been proposed that this pathway is a result of horizontal gene transfer (HGT) between *E. huxleyi* and their coccolithoviruses [1,9,39]. Similar HGT events with far-reaching ecological relevance and consequences have been reported between other marine viruses and their hosts. These include photosynthesis-related genes such as photosystem II core reaction center protein D1 and a high-light-inducible protein in *Prochlorococcus* phages [41], as well as phosphate transporter genes (e.g., *pho4* and *phoH*) in viruses infecting *Bathycoccus*, *Micromonas* and *Ostreococcus* [42].

Since the sequencing of the EhV-86 genome, an additional 12 EhV strains have been isolated and their genomes sequenced [43,44,45,46,47,48,49]. This makes EhVs one of the largest collections of *Phycodnaviridae* viruses in culture with complete or near-complete genome sequences available. In this study, we conduct an in-depth comparison of these EhV genomes for the first time and consider the functional and ecological significance of variation observed between strains. Further, we investigate potential evolutionary links between these viruses and the other domains of life, in particular bacteria which co-occur with EhVs in the marine environment. Bacteria are important constituents of microbial food webs in marine habitats [50,51] and may themselves act as pathogens. As an example of the latter, certain bacteria have been found to induce caspase-like activity and cell death in *E. huxleyi* [52]. Diseases of macroalgae in coastal habitats have also been attributed to bacteria [53], which likely coexist with a diverse viral community [54]. Interestingly, bacterial-like genes have been reported in several *Phycodnaviruses*. For example, 48 to 57 bacterial-like genes were identified in three viruses of *Chlorella*, and 81% of the total bacterial-like genes identified in *Mimivirus* have no homologs in even distantly related eukaryotes [55,56]. The possibility that EhVs may also have acquired genes from marine microbes (other than their host) via HGT will be explored.

## 2. Methods

### 2.1. Coccolithoviruses and Their Phylogeny

The genomes of 13 EhV strains were retrieved from National Center for Biotechnology Information (NCBI) (Table 1). One genome (EhV-86) is complete. The remaining twelve are near-complete draft genomes which contain gaps between some of the contigs (see Section 3.3). A 14th genome (EhV-163) was not included in this analysis to avoid biases due to its incomplete sequence (80%) [43]. CLUSTALW multiple sequence alignment of two EhV genes, DNA polymerase and serine palmitoyltransferase (2921 and 2604 bp long, respectively), and subsequent phylogenetic analysis was conducted using MEGA6 [57]. The evolutionary history of these sequences was inferred using two independent methods: Neighbor-Joining and Maximum Likelihood [58].

### 2.2. Whole Genome Reconstruction, Alignment and Comparison

Annotated EhV genomes [1,44,45,46,47,48,49] were used to build genomic alignments. Prior to analysis, the gaps between the assembled annotated contigs of each draft genome were made the same length by adding a known length of ambiguous nucleotides (i.e., a series of 10 Ns). The Artemis Comparison Tool (ACT) was then used for BLASTn pairwise alignment and visualization of the draft genome sequences against the fully assembled non-gapped reference genome of EhV-86 [59]. This enabled identification of the most likely orientation and placement of contigs (note that inversions are common in virus genomes and it is possible that inversions represent a real difference between the genomes). Misplaced contigs were placed in their appropriate location and converted into the correct orientation manually. Subsequently, MAUVE 2.0, a program for the identification of conserved genomic DNA regions and rearrangements, was used to elucidate the variable regions of each genome [60] in relation to EhV-86. Additional gene prediction analysis and functional annotations were performed within the publically available online Integrated Microbial Genomes—Expert Review platform (IMG/ER; [61]). Pairwise average nucleotide identity between each pair of genomes was also performed in this platform using Average Nucleotide Identity (ANI) analysis [62,63]. Briefly, ANI was used to compare all protein coding genes within each EhV genome to the non-gapped reference genome of EhV-86 (Table 2). Only bidirectional best hits (BBHs) with >70% sequence identity over >70% of the length of the shorter sequence in each BBH pair were retained.

### 2.3. “Best BLAST Hit” Analysis of Genomes

A “Best BLAST hit” analysis was performed on the predicted CDSs of the EhV genomes using a BLASTp algorithm [64] with default parameters against the publically available NCBI non-redundant protein database, excluding viruses. The “Best BLAST hit” analyses used a comprehensive list of viral genes (in this case all the predicted CDSs of 13 EhV genomes) as a query against the NCBI non-redundant protein sequences and captured the top-hit taxonomic data if available (e.g., Domain, Phylum, Class, Order, Family, Genus, Species), associated with the subject homolog (Target), as well as sequence match scoring parameters. An initial search pulled all hits. These were clustered according to “Domain” and evaluated for alignment and Bit scores, which were normalized to the scoring system. While we recognize that there is no perfect cut-off, we have chosen to limit the dataset to Bit scores above 50 for “Domain”, and above 100 (E-value < 1 × 10^−19^) for the subsequent taxon categories. These analyses fall within the suggested boundaries [65].

Specific BLASTp analysis of each EhV genome against the pan genome of *E. huxleyi* CCMP1516 [66] and against other EhVs was conducted within the IMG/ER platform using a cut-off E-value of < 1 × 10^−5^ and identity of >50%. Additional BLASTp analysis of EhV genes highly similar to *E. huxleyi* CCMP1516 host genes and those similar to other putative genes in other taxa discovered during the “Best BLAST hit” analysis (see Section 3.5 and Section 3.6) were performed within NCBI against the non-redundant protein sequence database that includes viruses (Tables S1–S10).

## 3. Results and Discussion

### 3.1. Phylogenetic Relationships of Coccolithoviruses

**Figure 1:**
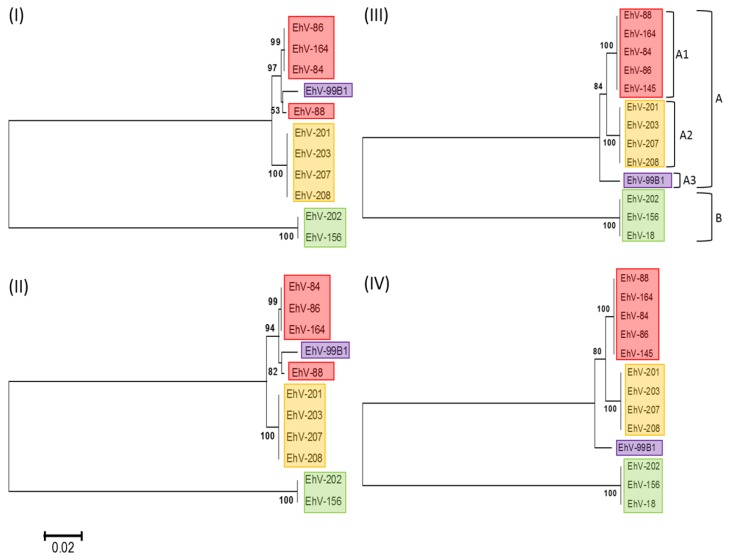
Phylogenetic analysis of coccolithoviruses based on their DNA polymerase and serine palmitoyltransferase (SPT) genes. The evolutionary history of 13 EhV strains was inferred based on the 2604 bp long SPT (I and II) and 2921 bp long DNA polymerase (III and IV) genes, using the Neighbor-Joining (I and III) and Maximum Likelihood (II and IV) methods. Note that EhV-18 and EhV-145 are absent from the serine palmitoyltransferase tree due to the full length SPT protein being split over two separate genes in their respective genomes. Based on the DNA polymerase phylogeny, the EhVs cluster into two main clades: A and B (green). Clade A is further divided into sub-clusters A1 (red), A2 (yellow), and A3 (purple). The percentage of replicate trees in which the associated taxa clustered together in the bootstrap test (1000 replicates) are shown next to the branches. The evolutionary distances were computed using the Tamura-Nei method and are in the units of the number of base substitutions per site.

Phylogenetic analysis of available DNA polymerase gene sequences, one of the commonly used phylogenetic markers for the dsDNA viral kingdom, indicated that the EhVs cluster into two major groups: A and B (Figure 1). The division into these groups was primarily due to a longer DNA polymerase gene present in EhV-18, EhV-156 and EhV-202 (caused by an insert of 12 bp, which results in the addition of glycine, threonine and two prolines at the end of the coding region). Group A was divided into three sub-clusters, where cluster A1 represents strains isolated primarily from the English Channel in 1999, A2 represents strains isolated primarily from the English Channel in 2001, and A3 represents a single strain isolated in 1999 from a Norwegian fjord. As such, these sub-clusters appear to be correlated with both year of isolation and location of origin. However, several exceptions were identified: EhV-145 and EhV-164 also clustered with A1 but were isolated after 1999. EhV-202, isolated in 2001 from the English Channel, clustered in B, with EhV-156 and EhV-18 (due to the 12 bp insertion mentioned previously), rather than A2. To further test these EhV groupings, phylogenetic analysis was also performed using the less conserved serine palmitoyltransferase (SPT) gene. This revealed similar groupings, with the exception of EhV-99B1 which clustered with A1.

The phylogenetic clades were also confirmed by the ANI analysis, in which the closest relative to EhV-86 based on the bidirectional pairwise alignment of the total number of genes was EhV-164 (placed in sub-clade A1), whereas those the furthest away were EhVs in clade B (Table 2).

Interestingly, significant differences have been observed in the infection dynamics of representative EhVs belonging to each phylogenetic clade identified in this study (Figure 1), both in respect to their lytic period [13] and their ability to utilize their host for the production of virally encoded glycosphingolipids, which are crucial for the successful demise of host cells [67].

### 3.2. Homology, Heterology and Genome Structure

**Figure 2:**
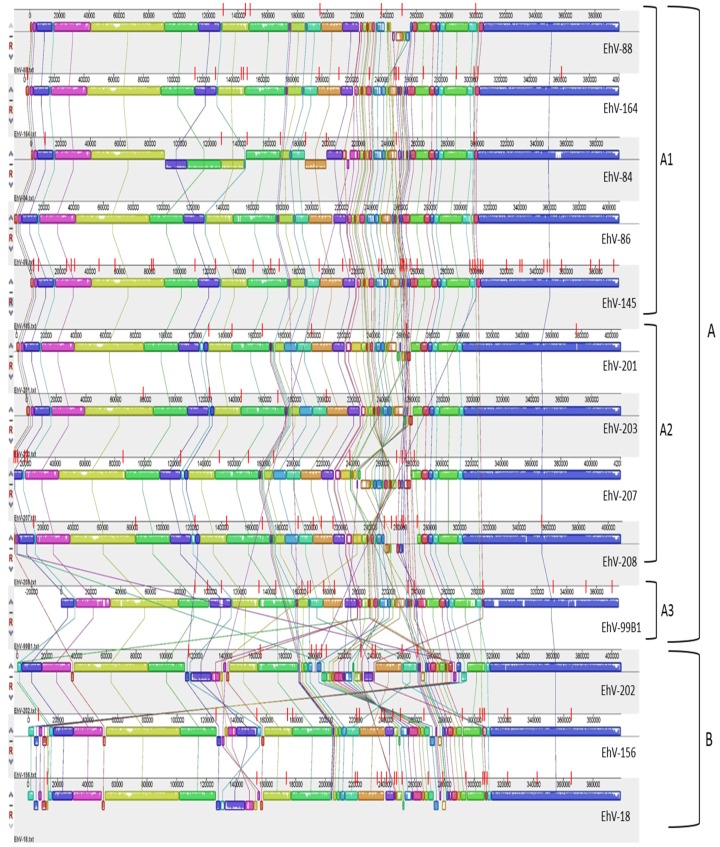
Whole genome alignment of sequenced coccolithovirus genomes. The genomes were aligned using MAUVE, in relation to the non-gapped backbone genome of EhV-86. Syntenous blocks are indicated in the same colours and the lines that connect them indicate the position of each block in relation to the same block of genes in the genome of EhV-86. The small red lines on each genome represent the exact positions of the gaps that separate the different contigs within each draft genome. The genomes are ordered based on their DNA polymerase phylogeny (Figure 1), based on the ANI analysis of this study (Table 2), and based on previously published microarray data that puts them into the aforementioned groups and sub-clades [20].

Whole genome alignment revealed that the EhV genomes were syntenic with the exception of a ~80,000 bp section located in the middle of each genome (Figure 2) enriched with early transcription promoter elements [1,20,68]. As expected [37], this hyper-variable region was characterized by numerous inversions, rearrangements and base pair substitutions. One possibility is that the observed variation in this region is a result of the gaps in the draft genomes between the different contigs (Figure 2). Although this may be the case for some of the draft genomes (i.e., EhV-18, EhV-156, EhV-202, and EhV-208), where the highest number of gaps are in that region, it is not for others. For example, EhV-201 and EhV-99B1 sequences only have 2–3 gaps in that region, yet still exhibit variability. Further, many gaps are located in areas outside this region and show little variation; the draft genome of EhV-145 has 10 gaps in the section that ranges from ~300,000 bp to ~397,000 bp, yet variation in this section is minimal. It is therefore likely that the early transcriptional profile of these viruses can differ considerably.

The misalignment of different sections within the hyper-variable region (evident by numerous crossing lines between the co-linear blocks in Figure 2) becomes more prominent the further a genome is from those in cluster A1 (Figure 2). This is consistent with the DNA polymerase based phylogeny (Figure 1), in which viruses belonging to group B are more distant from those in group A1. Considered together, the genome alignments (Figure 2), and ANI analysis (Table 2) support the clustering of EhVs into the aforementioned groups and is consistent with DNA polymerase and SPT based phylogenies.

### 3.3. Genome Size and Putative Gene Differences

Previous pulsed-field gel electrophoresis (PFGE) of coccolithovirus genomes indicates that they have an average size of 410 kb [2]. In our study, the estimated length of the EhV genomes ranged from 376,759 to 421,891 bp (with a GC content of 39.94% to 40.49%), suggesting considerable variability in EhV genome size. For example, EhV-99B1 appears significantly shorter than the rest of the genomes (Figure 2). The genomes also differed in the number of predicted protein coding sequences (CDSs). They contained between 444 and 548 CDSs, from which an average of 90 had functional prediction, including three to six transfer RNA (tRNA) genes (Table 3). Due to the highly repetitive nature of the hyper-variable region, some of the variations observed could be attributed to sequencing errors, misalignments introduced during the original genome assembly, and the numerous gaps between the different contigs. However, these variations may also reflect real differences, as it is unlikely that regions as big as 45,132 bp long (the difference between the longest and shortest genome) were incorrectly sequenced or not included in the assemblies. These variations may have real consequences on the virus life cycle, affecting the time for viral genome synthesis within infected cells and, to an extent, the size of the capsid into which the DNA will be packed. As viruses exploit the resources of the host cell to synthesize new, infectious viral particles, these inherent differences may ultimately affect the number of viable viruses produced during infection.

Although strain-specific differences in the number and composition of transfer RNAs (tRNAs) encoded within the genomes were identified, the tRNAs for Arg, Asn and Gln were common to all (Table 4). Similarly, Pagarete et al. [44] report differences in the type of tRNAs encoded by EhV-86 and EhV-99B1. However, after an in-depth study of the different codon frequencies of the genomes of EhV-86 and EhV-99B1, they concluded that the difference in tRNAs does not correspond to the respective codon frequencies in each genome and that it is more likely that strain-specific tRNA differences represent adaptations to different host strains. If so, viruses with a larger number of tRNAs may have an advantage in terms of host range and translation within different host genotypes. Allen et al. [20] observe (in the absence of statistically robust data) that out of 23 different host strains tested, EhV-207 (which codes for six tRNAs) infects 10, whereas EhV-86 (which codes for five tRNAs) infects eight. Moreover, EhV-207 outcompetes EhV-86 when placed in direct competition over a single host genotype and has a higher rate of virus particle production [13]. The extent to which tRNAs are responsible for these observations has yet to be determined.

Most predicted CDSs were shared between all EhV genomes but a number of strain-specific CDSs were detected (Table 5). EhV-84 and EhV-202 contain the largest number of potentially “unique” genes (27 and 18, respectively). This is intriguing, as the other genomes in clade A1 and B (to which EhV-84 and EhV-202 belong respectively) do not exhibit a large number of “unique” genes (Table 5).

The observed strain-specific genomic differences within members of the same clade (Table 3, Table 4 and Table 5) highlight the limitations of virus taxonomy using a single gene such as DNA polymerase, when one tries to infer upon the evolutionary connections of these large viruses, the origin of certain genes, and potentially their functional implications. These limitations also become apparent in large-scale phylogenetic studies that include numerous taxa across the domains of life. Almost two decades ago, Villarreal and DeFilippis [69] suggested an alternative hypothesis for the flow of genetic material, with an emphasis on the virus DNA polymerase. Based on a large number of hits of virus DNA polymerases to other taxa, they suggest that algal viruses did not acquire replication genes from their eukaryotic hosts but vice versa [69]. We performed a similar analysis using the DNA polymerase gene of EhV-86 against the non-redundant protein sequence database in NCBI. The closest hit was to a DNA polymerase of the Yellowstone lake phycodnavirus 1 (reference sequence: YP_009174732.1), with a query cover of 81%, E-value of 9 × 10^−137^ and identity of 35% (Table S1), other *Phycodnaviruses*, and other organisms such as *Klebsormidium laccidum* (which belongs to a genus of filamentous charophyte green algae).

### 3.4. Coccolithovirus Gene Similarities to the Emiliania huxleyi Host

The prevailing dogma is that many EhV genes were acquired from *Emiliania huxleyi* through HGT. BLASTp analysis of EhV genomes revealed that, on average, ~25 EhV protein coding sequences were highly similar to counterparts in the host genome of *E. huxleyi* CCMP1516 (consistent with previous analysis of this host genome [66]). Unfortunately, most of these genes have no assigned function yet. Those with a predicted function include a group of genes that encode a de novo sphingolipid biosynthesis pathway (i.e., serine palmitoyltransferase [45%], sterol desaturase [42%], transmembrane fatty acid elongation protein [56%], and lipid phosphate phosphatase [29%]). BLASTp analysis of the EhV serine palmitoyltransferase gene (which encodes the rate-limiting enzyme in this pathway) showed that the most similar genes in the non-redundant protein sequence database of NCBI are putative serine palmitoyltransferases in *E. huxleyi*, *Chrysochromulina* sp. and *Perkinsus marinus* (Table S2). These genes are essential for the progression of infection through the production of virus-derived glycosphingolipids [28] and induction of PCD pathways within infected cells [17], and are present in all EhV genomes analysed. As previously reported [49], the two protein subunits of SPT (LCB1 and LCB2) are encoded by two separate genes in the genomes of EhV-18 and EhV-145. This separation over two genes may reflect the ancestral form prior to the fusion of the two subunits into a single gene (as seen in all other EhVs). The functional implications of this during infection are so far unknown. Nevertheless, the acquisition of this near complete pathway represents a classical example of HGT of a virus with its host, and illustrates a finely tuned co-evolutionary relationship between a host and its virus. In this case, it is manifested by a control for sphingolipid biosynthesis [29].

With the exception of EhV-99B1, EhV-202, EhV-18 and EhV-156, the CDS with the highest identity (i.e., 86.4%) to a similar gene in *E. huxleyi* CCMP1516 is a putative phosphate permease transporter (denoted as *ehv117* in EhV-86). This transporter is encoded by all EhV isolates from the English Channel and the Scottish coast, but absent in the Norwegian isolate EhV-99B1 ([44]; and this study) and the partially sequenced genome of the Norwegian isolate EhV-163 (not included in this study) [43]. Instead, a 75-bp scar remnant of the transporter gene is still present at the 3′ end of this gene in both Norwegian isolates, and is replaced by a putative endonuclease [43]. This indicates that the Norwegian fjord EhVs once possessed the gene [44] and have since lost it. Such genes are common in *E. huxleyi*, which has six inorganic phosphate transporters and an alkaline phosphatase that enable it to thrive in low phosphorous conditions [66]. Phosphorus is also essential for successful viral replication in most host-virus systems [70,71,72]. However, we do not currently know if the *ehv117* gene product is used to enhance phosphate acquisition from the external environment during infection. Its replacement with an endonuclease encoding gene in EhV-99B1 is intriguing, as this may function to provide additional phosphate via utilization of internal cellular resources, rather than acquisition of external phosphate via transporter activity. This could reflect the selective pressure imposed by particular geographic locations that are characterized by specific physicochemical conditions. For instance, the frequency of phosphate uptake genes in metagenomic datasets is higher in locations where the average phosphate concentration is lower (e.g., North Atlantic Gyre) [42,73]. Like SPT, it is most similar to a homologue in *E. huxleyi* (Table S3) and was likely acquired by HGT with its host. Other high hits in NCBI include similar genes in *Ostreococcus lucimarinus* and *Ectocarpus siliculosus*. Similarly, a phosphate transporter gene (i.e., *pho4*) was identified in the viruses infecting *Ostreococcus* and it was proposed that this was also a result of HGT [42].

For EhVs where the *ehv117* transporter gene is absent, the CDS with the highest percentage similarity to a CDS in *E. huxleyi* CCMP1516 varies. In EhV-99B1, it is a predicted ribonucleoside-diphosphate reductase protein (RNR) (71.9% similar) whereas in EhV-202, EhV-18, and EhV-156 it is a predicted polyubiquitin protein (94.7% similar). While the former is an enzyme crucial for the conversion of ribonucleotides to deoxyribonucleotides for DNA synthesis, the latter is involved in protein degradation. We cannot confirm if these genes are also a result of HGT with their host, as there were many top hits in the NCBI non-redundant protein sequence database to other taxa, including the haptophyte *Chrysochromulina*, *Chitinophaga niabensis* bacteria, and the Gram-negative bacteria *Cnuella takakiae*, in the case of RNR (Table S4), and radiolarians such as *Collozoum inerme*, and protists such as *Vitrella brassicaformis*, in the case of polyubiquitin (Table S5).

### 3.5. Coccolithovirus Gene Similarities to Other Eukaryotes

**Figure 3:**
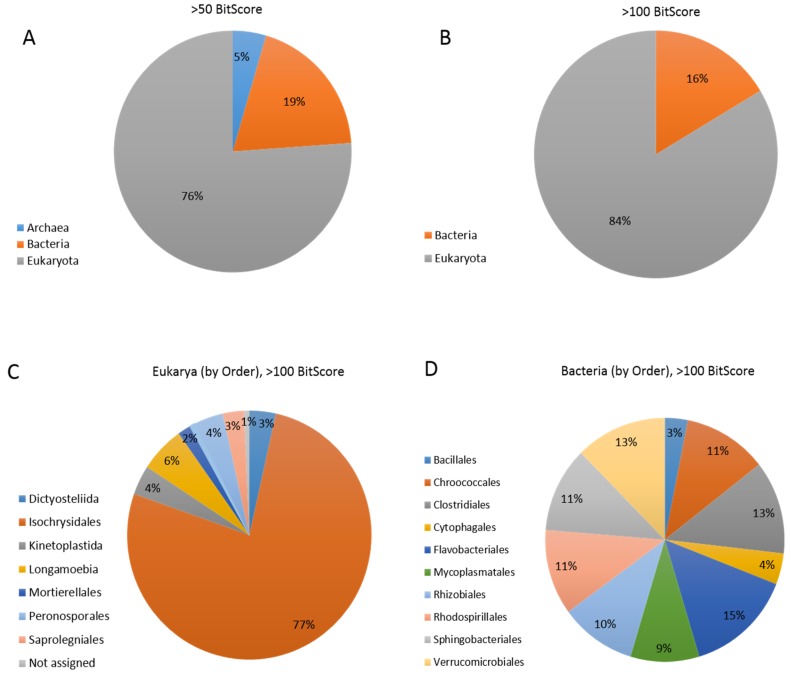
“Best BLAST hit” analysis of coccolithovirus CDSs in relation to the three domains of life: Eukarya, Bacteria and Archaea. Predicted genes within EhV genomes were BLASTp analyzed against possible hits in the three domains of life using a gene BitScore of >50 (**A**); and >100 (**B**). Further EhV gene hits analysis to the taxonomic level of “order” in Eukarya (**C**); and Bacteria (**D**) was performed using a BitScore of >100.

As previously mentioned, the current consensus is that many EhV genes are a result of HGT between coccolithoviruses and their specific host [39]. If this is true, it demonstrates that these viruses are entwined within the molecular workings of their hosts and have managed to acquire genes that target and coerce core aspects of their cellular metabolism. Alternatively, many EhV genes (including some of those mentioned in Section 3.4) may have an alternative origin. Thirty-three percent of the total EhV gene hits in the “Best BLAST hit” analysis (which excluded viruses) matched Eukaryotes other than *Isochrysidales* (the order to which *E. huxleyi* belongs) (Figure 3). These genes included those involved in nucleotide transport and metabolism, replication, recombination and repair, and transcription (Table 6). Among these other Eukaryotes are phylogenetically distant organisms such as moulds, fungi, unicellular flagellated protozoa, centric diatoms, amoeba, and green algae. Most of these can be found in the planktonic realm where *E. huxleyi* also dwells, particularly diatoms whose development is often tightly linked to *E. huxleyi* growth in ecological successions [74]. In this study, some of the top EhV gene hits against other genera include DNA-directed RNA polymerase subunit-B (an enzyme that produces mainly RNA transcripts) and DNA ligase (an enzyme that facilitates the joining of DNA strands) (Table 6). The former had top hits against members of the genus *Dictyostelium*, a group of soil living amoeba, whereas the latter had top hits against *Micromonas*, a group of chlorophyte green algae (Tables S6 and S7).

It is also plausible that EhVs have, or have had in their evolutionary past, alternative hosts that have enabled them to facilitate HGT with other taxa. Some giant algal viruses have the capacity to alternate between hosts belonging to different genera. For example, viruses capable of infecting *Haptolina hirta* infect also *Prymnesium kappa* and vice versa; [75]). Alternatively, there may have been a retroviral stage during the evolution of the *E. huxleyi*-virus system. Several EhV genes thought to have originated via HGT with *E. huxleyi* (including those for the de novo sphingolipid biosynthesis) lack introns, unlike their counterparts in the host genome. This suggests the possible involvement of coccolithophore-infecting RNA viruses in mediating HGT, although such viruses have not been observed yet.

### 3.6. Coccolithovirus Gene Similarities to Bacteria

There are close ecological relationships between *E. huxleyi* and some bacterial groups. A previous study reports that *E. huxleyi* blooms in the North Atlantic are dominated by *Roseobacter*, SAR86, SAR11, and other *Alpha* and *Gammaproteobacteria* [76]. In their study, bacteria belonging to the *Roseobacter* genus and SAR86 and SAR11 clades account, together, for >50% of the bacterial rDNA in surface waters, whereas a cyanobacterium and members of the *Alphaprotobacteria* are associated with chlorophyll a-rich waters in the euphotic zone (0–50 m in depth), the typical niche at which coccolithophores are found [76]. A link between coccolithophores and bacteria is also found in cultures of *E. huxleyi* and *Coccolithus pelagicus*, which are enriched in hydrocarbon-degrading bacteria belonging to *Marinobacter* and *Marivita* [77]. These bacteria can colonize exopolymeric substances exuded by microalgae, but the ecological role of this phenomenon is unknown [78]. This tight evolutionary coexistence involving *E. huxleyi*, its viral community, and associated bacteria may have resulted in HGT across the domains.

In our more conservative “Best BLAST hit” analysis (using a bit-score of >100) we saw that 16% of the EhV genes also shared strong similarities to bacterial genes (Figure 3), involved in nucleotide, lipid, and carbohydrate transport and metabolism; and replication, recombination and repair (Table 6). Interestingly, almost half of the bacterial-like genes in the Chlorella virus NY2A, and a fifth of those in Mimivirus were proposed to be involved in DNA replication and repair [55,56]. In our analysis, top hits to bacteria include genes that code for sialidase, deoxycytidylate deaminase and fatty acid desaturase. These hits were against members of the genus *Clostridium*, *Sphingobacterium* and *Niveispirillum* respectively (Table 6, and Tables S8–S10). Bacteria from these genera are consistently found in all coccolithophore cultures studied by Green et al. [77]. The hits against the *Sphingobacteriales* are particularly intriguing as members of this order are characterized by the presence of cellular lipid components that are comprised of high concentrations of sphingophospholipids [79]. The rate limiting step in sphingolipid biosynthesis in *S. multivorum* is serine palmitoyltransferase (SPT) [80], as in the *E. huxleyi*—virus system [81]. Although the EhV SPT is more similar to the host gene (as indicated above), the closest crystal structure in the PDB database to the EhV SPT is *Sphingobacterium multivorum* [67]. This structure was used to model the EhV derived SPT enzyme and to infer its catalytic capabilities during infection [36,67].

Moreover, in *S. multivorum*, sialidase specifically hydrolyzes deaminated neuraminic acid linkages and is localized in the periplasm [82,83]. Viruses such as influenza, adenoviruses, and polyomaviruses [84,85], can use virus-derived sialidase to target host sialic acid glycosphingolipids located on the host membrane. A similar process could be fundamental for EhV host recognition and entry, notably because susceptible *E. huxleyi* strains are enriched in glycosphingolipids (GSLs) with a sialic acid-modified glycosyl head-group [12,14]. These GSLs are proposed as a target for hydrolysis by EhV-encoded sialidases or as a ligand for the attachment of EhV lectin proteins [23]. Indeed, virus-derived sialidase transcripts are detected during EhV-86 infection 6–24 h post infection [21].

Surprisingly, we observed that the putative sialidase gene is severely truncated (up to 95% of its extension) in some EhV strains. Its full form is present in 1999 isolates (as well as EhV-164 and EhV-145), whereas those isolated in 2001 (as well as in EhV-18 and EhV-156) have a truncated form. In the genomes with the truncated form, the reduction is from a full gene of 1122 bp to 180 bp. Further reduction is seen in EhV-18 and EhV-156 where only a 60 bp fragment of the original gene remains. To date, EhV infectivity data do not show an association between the lack of a full length sialidase gene and a reduced ability to infect *E. huxleyi*. Hence, the selection against this gene in many EhVs and the fact that infection still occurs (presumably alongside other viruses able to utilize the active gene), suggests that its presence is not essential. Previous studies of Influenza A viruses show that a lack of functional sialidase is not essential to all viruses and that some viruses can adapt and grow in its absence in tissue cultures, mice, and embryonic eggs [86].

The role of two other bacterial-like genes, fatty acid desaturase and deoxycytidylate deaminase (Table 6) identified in EhVs requires further investigation. Fatty acid desaturases catalyze the desaturation of fatty acids. During EhV infection, a significant remodeling of the fatty acid profile of *E. huxleyi* occurs and this gene most likely plays an important role [30,87]. Deoxycytidylate deaminase is an enzyme involved in the biosynthesis of deoxyribonucleoside triphosphates (dNTPs). Its role, however, during infection remains elusive, as its expression has not been confirmed [1].

### 3.7. Possible Mode for HGT with Bacteria

Bacterial homologues in the genomes of nucleocytoplasmic large DNA viruses (NCLDVs) have been previously reported [55,56]. To our knowledge, our study is the first time that this is specifically identified in coccolithoviruses. While it is possible that some of the aforementioned genes were incorporated in the EhV genomes before the evolutionary divergence of eukaryotes and prokaryotes (a detailed “Best BLAST hit” analysis against ancestors of *E. huxleyi* such as *Tisochrysis lutea* and *Prymnesium parvum*, as well as against other primitive lineages of bacteria should reveal that), other explanations are also plausible. HGT could have occurred in situations where the genetic material of EhVs and bacteria co-occurred during virus replication. It is possible that EhVs and bacteria share, at moments, the same intracellular environment within *E. huxleyi*, particularly in light of recent data highlighting the importance of mixotrophy in phytoplankton [88]. For instance, in another NCLDV (i.e., *Cafeteria roenbergensis* virus), a 38-kb genomic region was suggested to be bacterial in origin and possibly acquired during an infection of its microflagellated grazer host that often contains a phagocytosed bacteria in its cytoplasm [89]. While there is no direct evidence for the engulfment of bacteria by *E. huxleyi*, a recent study of the different life stages of this alga shows that both diploid calcifying and motile haploid cells can engulf microbeads as big as 0.5 µm in diameter [90]. Also, a high abundance of transcripts linked to the digestive apparatus and those related to endocytosis are detected in diploid *E. huxleyi* cells, suggestive of an inherent ability of coccolithophores to participate in mixotrophy [90]. Endocytotic vesicles may be an additional mechanism for EhV infection [22], and *E. huxleyi* feeding on cohabiting bacteria might sometimes be infected by lytic viruses. Such mixotrophic-viral infection events may facilitate HGT across the domains.

Whether these events have occurred multiple times during which individual bacterial genes from different co-habiting bacteria were acquired by EhVs throughout their evolutionary history, or there have been only a few events during which large “chunks” of bacterial DNA (comprising of several adjacent genes, possibly sharing a similar function) were incorporated into EhVs, is unknown. However, the EhV genes with the highest bit-scores to Bacteria in our study were not genomically-located near each other in the analyzed genomes and did not appear to hit the same bacterial taxa. This is therefore consistent with the occurrence of multiple independent gene transfer events.

## 4. Conclusions

In light of the increasing body of literature that focuses on high-throughput metagenomics data and direct sequencing of virus genomes from environmental samples, it is important to remember the utility of model host-virus systems and single genome analysis. Here, we provided a review of the genomic features of 13 coccolithovirus strains and highlighted the differences and similarities in their respective genomes. We showed that the classification of coccolithoviruses using whole genome analysis mimics that of conserved gene based phylogeny. However, this approach does not allow the elucidation of functionally important differences among closely related viruses, and a detailed gene-by-gene examination may sometimes be more suitable. In addition, although many coccolithovirus genes were most similar to their known host and other eukaryotes, we highlighted the close and often ignored relationships that microalgae and their viruses have with bacteria in the marine environment. We revealed that for EhVs, as for other NCLDVs, HGT does not necessarily follow the traditionally expected pattern of host association. This analysis, performed across the three domains of life, contributes to some central questions in current viral ecology and evolution: (1) what defines a viral gene? (2) Where do viral genes come from? (3) are viruses able to serve as gene transfer agents across the domains of life? The existence of clear links to both Eukarya and Bacteria in this giant virus genomic assembly enhances the idea that viruses play a central role in the transfer of genetic information among the different life domains. Unwinding the relationships within the viral “domain”, understanding how it came to be, and how it has and continues to shape the three modern domains that we can easily recognize today remains a challenge.

## Figures and Tables

**Table 1:** A brief description of the coccolithoviruses used in this study, their geographical origin, year of isolation and sea water depth from which they were obtained.

Strain #	Isolate	Isolation	Lat/Long	Depth (m)	Date	NCBI	Reference *
	Location	Date			Sequenced	Accession #	
EhV-84	E.C.	1999	50°15′ N, 04°13′ W	15	2011	JF974290	Schroeder et al., 2002 [2]; Nissimov et al., 2011 [45]
EhV-86	E.C.	1999	50°30′ N, 04°20′ W	surface	2005	AJ890364	Schroeder et al., 2002 [2]; Wilson et al., 2005 [1]
EhV-88	E.C.	1999	50°15′ N, 04°13′ W	5	2011	JF974310	Schroeder et al., 2002 [2]; Nissimov et al., 2012 [46]
EhV-201	E.C.	2001	49°56′ N, 04°19′ W	2	2011	JF974311	Schroeder et al., 2002 [2]; Nissimov et al., 2012 [46]
EhV-202	E.C.	2001	50°00′ N, 04°18′ W	15	2011	HQ634145	Schroeder et al., 2002 [2]; Nissimov et al., 2012 [47]
EhV-203	E.C.	2001	50°00′ N, 04°18′ W	15	2011	JF974291	Schroeder et al., 2002 [2]; Nissimov et al., 2011 [48]
EhV-207	E.C.	2001	50°15′ N, 04°13′ W	5	2011	JF974317	Schroeder et al., 2002 [2]; Nissimov et al., 2012 [46]
EhV-208	E.C.	2001	50°15′ N, 04°13′ W	5	2011	JF974318	Schroeder et al., 2002 [2]; Nissimov et al., 2012 [46]
EhV-99B1	N.F.	1999	60°20′ N, 05°20′ E	surface	2013	FN429076	Pagarete et al., 2013 [44]
EhV-18	E.C.	2008	50°15′ N, 04°13′ W	surface	2013	KF481685	Nissimov et al., 2014 [49]
EhV-145	Loss.	2008	57°72′ N, 03°29′W	surface	2013	KF481686	Nissimov et al., 2014 [49]
EhV-156	E.C.	2009	50°15′ N, 04°13′ W	surface	2013	KF481687	Nissimov et al., 2014 [49]
EhV-164	SSF.	2009	56°26′ N, 02°63′ W	surface	2013	KF481688	Nissimov et al., 2014 [49]

* The references refer to literature that first presented information on each isolate or its genome. E.C.: English Channel; N.F.: Norwegian Fjord; Loss: Lossiemouth (off the UK coast); SSF.: Scottish Shore of Fife (UK).

**Table 2:** Average Nucleotide Identity (ANI) analysis of EhV genomes against EhV-86. The analysis included 12 draft EhV genomes, where a higher ANI score indicates greater genome similarity.

Reference Genome	Draft Genome	ANI Score	Total BBH *	Clade
EhV-86	EhV-164	99.95	443	A1
	EhV-145	99.93	456	A1
	EhV-84	99.07	434	A1
	EhV-88	98.96	442	A1
	EhV-99B1	98.23	421	A3
	EhV-208	96.78	399	A2
	EhV-207	96.67	411	A2
	EhV-201	96.6	399	A2
	EhV-203	96.6	402	A2
	EhV-18	79.52	307	B
	EhV-202	79.42	312	B
	EhV-156	79.4	308	B

* BBH: bidirectional best hit.

**Table 3:** Predicted genomic characteristics of sequenced coccolithoviruses. The statistics for each genome were obtained from the annotated ordered genomes uploaded into the Integrated Microbial Genomes—Expert Review (IMG/ER) online genome analysis pipeline [66]. Note that the numbers of genes, bases, coding sequences (CDSs), coding bases and transfer RNAs (tRNAs) here are underestimates (except for EhV-86) due to incomplete genome sequences.

Genome Name	Genes	Total Bases	CDS	Coding Bases	Genes with Function Prediction	tRNAs	GC (%)	Number of Gaps in Genome
*E. huxleyi* virus 84	486	396620	482	334463	85	4	40.17	8
*E. huxleyi* virus 86	478	407339	472	369157	90	5	40.18	0
*E. huxleyi* virus 88	480	397298	475	357803	90	5	40.18	7
*E. huxleyi* virus 201	457	407301	451	363714	89	6	40.46	6
*E. huxleyi* virus 202	488	407516	485	352215	93	3	40.3	11
*E. huxleyi* virus 203	470	400520	464	364178	91	6	40.12	5
*E. huxleyi* virus 207	479	421891	473	371313	93	6	40.49	15
*E. huxleyi* virus 208	461	411003	455	348386	90	6	40.42	16
*E. huxleyi* virus 99B1	451	376759	444	333400	90	6	40.04	16
*E. huxleyi* virus 18	508	399651	503	346161	91	5	40.49	21
*E. huxleyi* virus 145	552	397508	548	350414	103	4	39.94	41
*E. huxleyi* virus 156	498	399344	493	351083	88	5	40.47	19
*E. huxleyi* virus 164	514	400675	510	354290	95	4	40.11	17

**Table 4:** Genes predicted to encode tRNAs in the genomes of 13 coccolithoviruses. Their presence (+) in each genome is indicated, and in grey shaded cells are those tRNAs common to all genomes. The phylogenetic clade of each genome (based on their DNA polymerase gene) is indicated above the column headers (see Figure 1). It is important to note that some of these tRNAs may still be present in the genomes of some EhVs in the unsequenced parts between the different contigs.

Phylogenetic Group		A										B		
		A1					A2				A3			
tRNA	Genome	EhV-84	EhV-86	EhV-88	EhV-164	EhV-145	EhV-201	EhV-203	EhV-207	EhV-208	EhV-99B1	EhV-18	EhV-156	EhV-202
Arg		+	+	+	+	+	+	+	+	+	+	+	+	+
Asn		+	+	+	+	+	+	+	+	+	+	+	+	+
Gln		+	+	+	+	+	+	+	+	+	+	+	+	+
Glu							+	+	+	+				
Ile		+	+	+	+	+					+			
Leu			+				+	+	+	+	+	+	+	
Lys				+			+	+	+	+	+	+	+	

**Table 5:** Coccolithovirus strain-specific CDSs that are not shared among the different viruses and are “unique” to each strain. Analysis was done using the “build in BLASTp” algorithm in IMG/ER [61] using a maximum E-value of 1 × 10^−5^ and a minimum % identity of 30. The phylogenetic clades regrouping genomes are shown above the column headers (see Figure 1).

Phylogenetic Group	A										B		
	A1					A2				A3			
Predicted CDS	EhV-84	EhV-86	EhV-88	EhV-145	EhV-164	EhV-201	EhV-203	EhV-207	EhV-208	EhV-99B1	EhV-202	EhV-18	EhV-156
hypothetical protein	27	3	4	7	8	4	4	9	9	6	18	9	6
putative endonuclease										2			
putative membrane protein		2		1	1					4			
putative transposase										1			
putative DUF814 domain containing protein										1			
zinc finger protein							1						
putative ribonuclease												1	1
glycosyltransferase family 29 (sialyltransferase)												1	1
**Total**	27	5	4	8	9	4	5	9	9	15	18	11	8

**Table 6:** “Best BLAST hit” analysis of EhVs against the Bacterial and Eukaryotic domains of life, as well as against the known host *Emiliania huxleyi*. The top EhV genes with function predictions are shown based on the “Best BLAST hit” analysis using a BitScore of >100 and are ordered based on their E-value. The COG (Clusters of Orthologous Groups of genes with similar functions across the domains of life) cluster for each gene was identified on IMG/ER, where the different letters represent metabolic pathways involved in nucleotide transport and metabolism [F]; carbohydrate transport and metabolism [G]; general function prediction only [R]; replication, recombination and repair [L]; lipid transport and metabolism [I]; transcription [K]; inorganic ion transport and metabolism [P]; intracellular trafficking, and secretion and vesicular transport [U].

**Major Hits against Eukarya**								
**Ehv Predicted Gene**	**COG ID**	**Identity (%)**	**E-Value**	**Bit-Score**	**Phylum**	**Class**	**Order**	**Genus**
DNA-directed RNA polymerase subunit B	K	40.39	0	825	NA	NA	*Dictyosteliida*	*Dictyostelium*
DNA ligase	L	51.29	0	612	*Chlorophyta*	*Mamiellophyceae*	*Mamiellales*	*Micromonas*
DNA topoisomerase	L	33	0	586	*Microsporidia*	NA	NA	*Nematocida*
DNA-dependent RNA polymerase II largest subunit	K	34.09	7 × 10^−171^	555	*Ascomycota*	*Sordariomycetes*	*Xylariales*	*Eutypa*
thymidylate synthase	F	49.7	1 × 10^−168^	497	NA	NA	*Peronosporales*	*Phytophthora*
DNA polymerase delta catalytic subunit	L	35.11	2 × 10^−132^	436	*Arthropoda*	*Malacostraca*	*Decapoda*	*Procambarus*
DNA helicase	L	49.3	3 × 10^−137^	419	*Bacillariophyta*	*Coscinodiscophyceae*	*Thalassiosirales*	*Thalassiosira*
**Major Hits against Bacteria**								
deoxycytidylate deaminase	F	62.96	8 × 10^−64^	206	*Firmicutes*	*Clostridia*	*Clostridiales*	*Clostridium*
Sialidase	G	30.03	2 × 10^−37^	148	*Bacteroidetes*	*Sphingobacteriia*	*Sphingobacteriales*	*Sphingobacterium*
DNA-binding protein	R	34.36	7 × 10^−32^	129	*Verrucomicrobia*	NA	NA	NA
endonuclease	L	45.67	4 × 10^−32^	121	*Proteobacteria*	*Alphaproteobacteria*	*Rhizobiales*	*Pseudochrobactrum*
fatty acid desaturase	I	34.76	2 × 10^−28^	121	*Proteobacteria*	*Alphaproteobacteria*	*Rhodospirillales*	*Niveispirillum*

NA: NCBI classification was not available for Phylum, Class, Order or Genus.

## Supplementary Materials

The following are available online at http://www.mdpi.com/1999-4915/9/3/52/s1, Table S1: BLASTp analysis of the EhV DNA polymerase gene against the non-redundant protein database on NCBI, Table S2: BLASTp analysis of the EhV DNA serine palmitoyltranferase gene against the non-redundant protein database on NCBI, Table S3: BLASTp analysis of the EhV phosphate permease gene against the non-redundant protein database on NCBI, Table S4: BLASTp analysis of the EhV ribonucleoside-diphosphate reductase gene against the non-redundant protein database on NCBI, Table S5: BLASTp analysis of the EhV DNA polyubiquitin gene against the non-redundant protein database on NCBI, Table S6: BLASTp analysis of the EhV DNA-directed RNA polymerase subunit B gene against the non-redundant protein database on NCBI, Table S7: BLASTp analysis of the EhV DNA ligase gene against the non-redundant protein database on NCBI, Table S8: BLASTp analysis of the EhV deoxycytidylate deaminase gene against the non-redundant protein database on NCBI, Table S9: BLASTp analysis of the EhV sialidase gene against the non-redundant protein database on NCBI, Table S10: BLASTp analysis of the EhV fatty acid desaturase gene against the non-redundant protein database on NCBI.
