# Supplementary material for: Coccolithoviruses: A Review of Cross-Kingdom Genomic Thievery and Metabolic Thuggery
Source: Viruses. 2017 Mar 18;9(3):52. doi: 10.3390/v9030052 (PMC5371807; doi:10.3390/v9030052)
Supplement: Supplementary file 1 [file viruses-09-00052-s001.docx]

| **Description** | **Max score** | **Total score** | **Query cover** | **E-value** | **Ident**  **(%)** | **Accession** |
| --- | --- | --- | --- | --- | --- | --- |
| putative DNA polymerase [Yellowstone lake phycodnavirus 1] | 442 | 442 | 0.81 | 9E-137 | 0.35 | YP_009174732.1 |
| hypothetical protein OlV2_240 [Ostreococcus lucimarinus virus 2] | 444 | 444 | 0.83 | 1E-136 | 0.35 | YP_009172731.1 |
| DNA polymerase [Ostreococcus tauri virus 2] | 441 | 441 | 0.83 | 1E-135 | 0.35 | YP_004063640.1 |
| putative DNA polymerase [Ostreococcus tauri virus OtV5] | 439 | 439 | 0.83 | 1E-134 | 0.35 | YP_001648316.1 |
| putative DNA polymerase [Yellowstone lake phycodnavirus 2] | 436 | 436 | 0.83 | 2E-134 | 0.36 | YP_009174650.1 |
| hypothetical protein OmV1_225 [Ostreococcus mediterraneus virus 1] | 438 | 438 | 0.83 | 2E-134 | 0.35 | YP_009172985.1 |
| putative DNA polymerase [Yellowstone lake phycodnavirus 2] | 434 | 434 | 0.83 | 4E-134 | 0.35 | YP_009174598.1 |
| hypothetical protein OtV6_228 [Ostreococcus tauri virus RT-2011] | 437 | 437 | 0.83 | 7E-134 | 0.35 | AFC35136.1 |
| DNA polymerase delta catalytic subunit [Procambarus clarkii] | 436 | 436 | 0.78 | 1E-133 | 0.35 | BAO20827.1 |
| DNA polymerase [Micromonas pusilla virus SP1] | 434 | 434 | 0.83 | 4E-133 | 0.35 | AET84947.1 |
| hypothetical protein BpV1_184 [Bathycoccus sp. RCC1105 virus BpV1] | 431 | 431 | 0.86 | 3E-132 | 0.33 | YP_004061614.1 |
| hypothetical protein OlV7_215 [Ostreococcus lucimarinus virus 7] | 432 | 432 | 0.83 | 4E-132 | 0.35 | YP_009173227.1 |
| hypothetical protein BpV2_189 [Bathycoccus sp. RCC1105 virus BpV2] | 430 | 430 | 0.83 | 6E-132 | 0.34 | ADQ91356.1 |
| DNA polymerase [Micromonas pusilla virus 12T] | 431 | 431 | 0.83 | 6E-132 | 0.35 | YP_007676285.1 |
| DNA polymerase delta catalytic subunit [Reticulitermes speratus] | 432 | 432 | 0.72 | 1E-131 | 0.36 | BAJ78756.1 |
| hypothetical protein OlV1_219 [Ostreococcus lucimarinus virus 1] | 429 | 429 | 0.83 | 9E-131 | 0.34 | YP_004061851.1 |
| DNA polymerase delta catalytic subunit [Tenodera aridifolia] | 430 | 430 | 0.82 | 9E-131 | 0.33 | BAJ78754.1 |
| DPOD1_ORYSA DNA polymerase delta catalytic subunit gb\|AAX96341.1\| dna pol (ISS) [Ostreococcus tauri] | 432 | 432 | 0.84 | 2E-130 | 0.35 | XP_003075187.1 |
| DNA polymerase delta catalytic subunit [Mus musculus] | 429 | 429 | 0.85 | 1E-129 | 0.32 | NP_035261.3 |
| DNA polymerase [Micromonas pusilla virus PL1] | 425 | 425 | 0.84 | 1E-129 | 0.35 | AET43521.1 |
| DNA-directed DNA polymerase [Mus musculus] | 429 | 429 | 0.85 | 2E-129 | 0.32 | CAA79895.1 |
| polymerase (DNA directed), delta 1, catalytic subunit, isoform CRA_e [Mus musculus] | 428 | 428 | 0.85 | 3E-129 | 0.32 | EDL22733.1 |
| polymerase (DNA directed), delta 1, catalytic subunit, isoform CRA_d [Mus musculus] | 428 | 428 | 0.85 | 4E-129 | 0.32 | EDL22732.1 |
| DNA polymerase delta catalytic subunit [Danaus plexippus] | 424 | 424 | 0.82 | 4E-129 | 0.34 | EHJ68324.1 |
| DNA polymerase delta catalytic subunit [Epiophlebia superstes] | 425 | 425 | 0.82 | 4E-129 | 0.34 | BAJ78739.1 |
| DNA-directed DNA polymerase, family B, pol2 [Ostreococcus tauri] | 429 | 429 | 0.84 | 5E-129 | 0.34 | CEG01117.1 |
| DNA polymerase delta catalytic subunit [Euconocephalus varius] | 426 | 426 | 0.83 | 6E-129 | 0.32 | BAJ78748.1 |
| polymerase (DNA directed), delta 1, catalytic subunit, isoform CRA_a [Mus musculus] | 427 | 427 | 0.85 | 8E-129 | 0.32 | EDL22729.1 |
| hypothetical protein LOTGIDRAFT_194456 [Lottia gigantea] | 426 | 426 | 0.72 | 1E-128 | 0.36 | XP_009062309.1 |
| DNA polymerase delta catalytic subunit [Panorpa takenouchii] | 426 | 426 | 0.82 | 1E-128 | 0.33 | BAJ78774.1 |
| DNA polymerase delta catalytic subunit [Metriocampa sp. 44] | 427 | 427 | 0.83 | 2E-128 | 0.33 | BAJ78732.1 |
| predicted protein [Ostreococcus lucimarinus CCE9901] | 423 | 423 | 0.7 | 2E-128 | 0.37 | XP_001416112.1 |
| PREDICTED: DNA polymerase delta catalytic subunit [Sorex araneus] | 426 | 426 | 0.89 | 3E-128 | 0.32 | XP_004619921.1 |
| hypothetical protein HELRODRAFT_91243 [Helobdella robusta] | 424 | 424 | 0.82 | 3E-128 | 0.33 | XP_009032004.1 |
| DNA polymerase delta catalytic subunit [Oratosquilla oratoria] | 422 | 422 | 0.82 | 3E-128 | 0.33 | BAO20826.1 |
| delta DNA polymerase [Fibulorhizoctonia sp. CBS 109695] | 424 | 424 | 0.84 | 4E-128 | 0.35 | KZP26619.1 |
| hypothetical protein SOVF_212670 [Spinacia oleracea] | 424 | 424 | 0.84 | 6E-128 | 0.35 | KNA03069.1 |
| DNA polymerase delta catalytic subunit [Gynaikothrips ficorum] | 424 | 424 | 0.83 | 8E-128 | 0.33 | BAJ78758.1 |
| DNA polymerase delta catalytic subunit [Daphnia magna] | 424 | 424 | 0.84 | 1E-127 | 0.34 | JAN33095.1 |
| unnamed protein product [Mus musculus] | 424 | 424 | 0.85 | 1E-127 | 0.32 | BAC40275.1 |
| DNA polymerase delta catalytic subunit [Salmo salar] | 424 | 424 | 0.83 | 1E-127 | 0.33 | NP_001167152.1 |
| Polymerase (DNA directed), delta 1, catalytic subunit [Mus musculus] | 424 | 424 | 0.85 | 2E-127 | 0.32 | AAH09128.1 |
| PREDICTED: DNA polymerase delta catalytic subunit [Peromyscus maniculatus bairdii] | 423 | 423 | 0.85 | 2E-127 | 0.32 | XP_006986263.1 |
| DNA polymerase delta catalytic subunit [Phraortes illepidus] | 423 | 423 | 0.82 | 2E-127 | 0.33 | BAJ78743.1 |
| PREDICTED: DNA polymerase delta catalytic subunit [Microtus ochrogaster] | 423 | 423 | 0.84 | 2E-127 | 0.32 | XP_005366922.1 |
| polymerase (DNA directed), delta 1, catalytic subunit, isoform CRA_b [Rattus norvegicus] | 422 | 422 | 0.85 | 3E-127 | 0.32 | EDM07475.1 |
| DNA polymerase delta' [Mesocricetus auratus] | 422 | 422 | 0.83 | 4E-127 | 0.32 | AAB47255.1 |
| PREDICTED: DNA polymerase delta catalytic subunit [Esox lucius] | 422 | 422 | 0.83 | 5E-127 | 0.33 | XP_010900491.1 |
| DNA polymerase delta catalytic subunit [Daphnia magna] | 422 | 422 | 0.84 | 6E-127 | 0.34 | JAN74322.1 |
| hypothetical protein K503DRAFT_769940 [Rhizopogon vinicolor AM-OR11-026] | 421 | 421 | 0.84 | 6E-127 | 0.34 | OAX38975.1 |

**Table S1 BLASTp analysis of the EhV DNA polymerase gene against the non-redundant protein database on NCBI.** The top 50 hits using the default BLASTp settings were pulled, and sorted based on the E value in an ascending order.

| **Description** | **Max score** | **Total score** | **Query cover** | **E-**  **value** | **Ident**  **(%)** | **Accession** |
| --- | --- | --- | --- | --- | --- | --- |
| serine palmitoyltransferase 1 [Emiliania huxleyi] | 623 | 836 | 88% | 0 | 44% | AID57147.1 |
| serine palmitoyltransferase [Chrysochromulina sp. CCMP291] | 585 | 804 | 79% | 0 | 43% | KOO34538.1 |
| serine palmitoyltransferase, putative [Perkinsus marinus ATCC 50983] | 526 | 627 | 79% | 9.00E-169 | 40% | XP_002771826.1 |
| serine palmitoyltransferase, putative [Perkinsus marinus ATCC 50983] | 525 | 613 | 86% | 4.00E-168 | 38% | XP_002774667.1 |
| Long chain base biosynthesis protein 2b [Symbiodinium microadriaticum] | 514 | 726 | 79% | 3.00E-157 | 38% | OLP82054.1 |
| serine palmitoyltransferase, putative [Entamoeba invadens IP1] | 428 | 428 | 80% | 4.00E-133 | 35% | XP_004254179.1 |
| serine palmitoyltransferase [Entamoeba histolytica HM-1:IMSS] | 427 | 427 | 88% | 1.00E-132 | 36% | XP_655300.1 |
| serine palmitoyltransferase [Galdieria sulphuraria] | 385 | 518 | 76% | 9.00E-120 | 44% | XP_005706634.1 |
| Long chain base biosynthesis protein [Zostera marina] | 375 | 465 | 79% | 2.00E-117 | 42% | KMZ59378.1 |
| hypothetical protein SELMODRAFT_108076 [Selaginella moellendorffii] | 365 | 365 | 46% | 4.00E-114 | 44% | XP_002978032.1 |
| PREDICTED: long chain base biosynthesis protein 2c [Oryza sativa Japonica Group] | 365 | 365 | 48% | 1.00E-113 | 42% | XP_015613384.1 |
| hypothetical protein SELMODRAFT_86377 [Selaginella moellendorffii] | 364 | 364 | 46% | 1.00E-113 | 44% | XP_002966707.1 |
| PREDICTED: long chain base biosynthesis protein 2a-like [Musa acuminata subsp. malaccensis] | 363 | 461 | 78% | 8.00E-113 | 41% | XP_009404745.1 |
| serine palmitoyltransferase, LCB2 subunit [Chondrus crispus] | 364 | 445 | 76% | 9.00E-113 | 40% | XP_005710134.1 |
| long chain base biosynthesis protein 2d [Zea mays] | 362 | 362 | 50% | 2.00E-112 | 41% | NP_001295432.1 |
| PREDICTED: long chain base biosynthesis protein 2d [Setaria italica] | 362 | 362 | 50% | 2.00E-112 | 41% | XP_004971156.1 |
| PREDICTED: long chain base biosynthesis protein 2a-like isoform X1 [Musa acuminata subsp. malaccensis] | 360 | 460 | 78% | 5.00E-112 | 41% | XP_009403557.1 |
| hypothetical protein SORBIDRAFT_03g044700 [Sorghum bicolor] | 360 | 360 | 50% | 8.00E-112 | 41% | XP_002459025.1 |
| PREDICTED: long chain base biosynthesis protein 2a [Brachypodium distachyon] | 360 | 360 | 50% | 9.00E-112 | 40% | XP_003577540.1 |
| PREDICTED: long chain base biosynthesis protein 2d [Brachypodium distachyon] | 360 | 360 | 50% | 9.00E-112 | 41% | XP_003564989.1 |
| hypothetical protein SETIT_001208mg [Setaria italica] | 359 | 359 | 49% | 1.00E-111 | 41% | KQL08449.1 |
| hypothetical protein SORBIDRAFT_05g018880 [Sorghum bicolor] | 359 | 359 | 50% | 1.00E-111 | 40% | XP_002450805.1 |
| hypothetical protein SOVF_110050 [Spinacia oleracea] | 359 | 359 | 50% | 1.00E-111 | 41% | KNA14161.1 |
| PREDICTED: long chain base biosynthesis protein 2d [Oryza sativa Japonica Group] | 359 | 359 | 50% | 2.00E-111 | 41% | XP_015613397.1 |
| Long-Chain Base 1 [Klebsormidium flaccidum] | 358 | 466 | 85% | 3.00E-111 | 41% | GAQ81699.1 |
| PREDICTED: long chain base biosynthesis protein 2d [Oryza brachyantha] | 358 | 358 | 50% | 3.00E-111 | 42% | XP_006645277.1 |
| serine palmitoyltransferase 2 [Thecamonas trahens ATCC 50062] | 358 | 462 | 78% | 4.00E-111 | 43% | XP_013758825.1 |
| PREDICTED: long chain base biosynthesis protein 2a [Musa acuminata subsp. malaccensis] | 358 | 450 | 78% | 6.00E-111 | 41% | XP_009419359.1 |
| PREDICTED: long chain base biosynthesis protein 2c-like [Oryza brachyantha] | 358 | 358 | 53% | 6.00E-111 | 41% | XP_006645276.1 |
| PREDICTED: long chain base biosynthesis protein 2a [Elaeis guineensis] | 357 | 357 | 50% | 8.00E-111 | 41% | XP_010907607.1 |
| PREDICTED: long chain base biosynthesis protein 2a-like [Elaeis guineensis] | 357 | 357 | 50% | 1.00E-110 | 41% | XP_010929641.1 |
| long chain base biosynthesis protein 2d [Aegilops tauschii subsp. tauschii] | 357 | 357 | 50% | 1.00E-110 | 41% | XP_020191116.1 |
| PREDICTED: long chain base biosynthesis protein 2a [Setaria italica] | 357 | 357 | 50% | 2.00E-110 | 40% | XP_004979342.1 |
| PREDICTED: long chain base biosynthesis protein 2a-like [Phoenix dactylifera] | 356 | 356 | 50% | 3.00E-110 | 41% | XP_008791061.1 |
| serine palmitoyltransferase 2 [Zea mays] | 356 | 356 | 50% | 3.00E-110 | 40% | NP_001148953.1 |
| PREDICTED: long chain base biosynthesis protein 2d-like [Musa acuminata subsp. malaccensis] | 356 | 356 | 51% | 4.00E-110 | 42% | XP_018678266.1 |
| PLP-dependent transferase [Coccomyxa subellipsoidea C-169] | 355 | 456 | 81% | 4.00E-110 | 41% | XP_005647033.1 |
| PREDICTED: serine palmitoyltransferase 2-like [Hydra vulgaris] | 357 | 460 | 86% | 6.00E-110 | 42% | XP_012555461.1 |
| PREDICTED: long chain base biosynthesis protein 2a-like [Musa acuminata subsp. malaccensis] | 355 | 355 | 52% | 7.00E-110 | 40% | XP_009411041.1 |
| long chain base biosynthesis protein 2a-like isoform X2 [Ananas comosus] | 355 | 355 | 50% | 7.00E-110 | 40% | XP_020112196.1 |
| Serine palmitoyltransferase 2 [Triticum urartu] | 355 | 355 | 49% | 8.00E-110 | 41% | EMS48138.1 |
| Long chain base biosynthesis protein 2a [Ananas comosus] | 355 | 355 | 50% | 8.00E-110 | 40% | OAY67492.1 |
| serine palmitoyltransferase [Lotus japonicus] | 355 | 355 | 50% | 9.00E-110 | 40% | BAC55228.1 |
| PREDICTED: long chain base biosynthesis protein 2b [Brassica napus] | 355 | 355 | 50% | 1.00E-109 | 40% | XP_013683201.1 |
| PREDICTED: long chain base biosynthesis protein 2b [Brassica rapa] | 354 | 354 | 50% | 1.00E-109 | 40% | XP_009150176.1 |
| uncharacterized protein LOC100285632 [Zea mays] | 354 | 354 | 50% | 1.00E-109 | 40% | NP_001151995.1 |
| PREDICTED: long chain base biosynthesis protein 2a-like [Malus domestica] | 354 | 354 | 53% | 1.00E-109 | 40% | XP_008379061.1 |
| PREDICTED: long chain base biosynthesis protein 2a [Phoenix dactylifera] | 354 | 354 | 50% | 2.00E-109 | 40% | XP_008775142.1 |
| PREDICTED: long chain base biosynthesis protein 2b-like [Brassica oleracea var. oleracea] | 354 | 354 | 50% | 2.00E-109 | 40% | XP_013630355.1 |
| PREDICTED: long chain base biosynthesis protein 2b-like isoform X2 [Brassica rapa] | 353 | 353 | 50% | 2.00E-109 | 40% | XP_018510150.1 |

**Table S2 BLASTp analysis of the EhV DNA serine palmitoyltranferase gene against the non-redundant protein database on NCBI.** The top 50 hits using the default BLASTp settings were pulled, and sorted based on the E value in an ascending order.

| **Description** | **Max score** | **Total score** | **Query cover** | **E-**  **value** | **Ident**  **(%)** | **Accession** |
| --- | --- | --- | --- | --- | --- | --- |
| phosphate repressible phosphate permease [Emiliania huxleyi] | 479 | 479 | 80% | 5.00E-162 | 60% | AAO15381.1 |
| PiT family transporter: phosphate [Ostreococcus lucimarinus CCE9901] | 446 | 446 | 96% | 8.00E-149 | 47% | XP_001422167.1 |
| high affinity phosphate transporter, probable [Ectocarpus siliculosus] | 443 | 443 | 96% | 7.00E-148 | 51% | CBJ33038.1 |
| high affinity phosphate transporter, probable [Ectocarpus siliculosus] | 439 | 439 | 99% | 3.00E-146 | 49% | CBJ33037.1 |
| hypothetical protein BpV1_203c [Bathycoccus sp. RCC1105 virus BpV1] | 437 | 437 | 96% | 9.00E-146 | 47% | YP_004061633.1 |
| PiT family transporter: phosphate [Ectocarpus siliculosus] | 436 | 436 | 98% | 3.00E-145 | 45% | CBJ32804.1 |
| PiT family transporter: phosphate [Ectocarpus siliculosus] | 436 | 436 | 97% | 5.00E-145 | 46% | CBJ32177.1 |
| high affinity phosphate transporter, probable [Ostreococcus lucimarinus CCE9901] | 436 | 436 | 96% | 7.00E-144 | 47% | XP_001416412.1 |
| Phosphate transporter [Ostreococcus tauri] | 431 | 431 | 96% | 1.00E-142 | 46% | CEG01038.1 |
| Pho4 high affinity phosphate transporter, probable (IC) [Ostreococcus tauri] | 431 | 431 | 96% | 4.00E-142 | 46% | XP_003075057.1 |
| hypothetical protein OlV2_256 [Ostreococcus lucimarinus virus 2] | 418 | 418 | 96% | 6.00E-139 | 46% | YP_009172747.1 |
| high affinity phosphate transporter [Tetraselmis chuii] | 417 | 417 | 96% | 2.00E-136 | 46% | AAO47330.1 |
| phosphate-repressible phosphate permease [Bathycoccus prasinos] | 413 | 413 | 96% | 7.00E-136 | 45% | XP_007514366.1 |
| putative inorganic phosphate transporter [Aureococcus anophagefferens] | 400 | 400 | 98% | 1.00E-130 | 45% | XP_009034232.1 |
| hypothetical protein AURANDRAFT_70513 [Aureococcus anophagefferens] | 385 | 385 | 97% | 9.00E-123 | 43% | XP_009032267.1 |
| putative inorganic phosphate transporter [Aureococcus anophagefferens] | 375 | 375 | 95% | 1.00E-120 | 42% | XP_009036803.1 |
| sodium/phosphate symporter [Blastocystis sp. ATCC 50177/Nand II] | 358 | 358 | 97% | 9.00E-115 | 40% | OAO11932.1 |
| high affinity phosphate transporter (ISS) [Ostreococcus tauri] | 351 | 351 | 84% | 2.00E-112 | 44% | XP_003075059.1 |
| hypothetical protein CHLNCDRAFT_33297 [Chlorella variabilis] | 353 | 353 | 96% | 8.00E-112 | 41% | XP_005843015.1 |
| high affinity phosphate transporter [Ectocarpus siliculosus] | 342 | 342 | 90% | 7.00E-109 | 42% | CBN77083.1 |
| hypothetical protein CHLNCDRAFT_141686 [Chlorella variabilis] | 347 | 347 | 96% | 8.00E-109 | 39% | XP_005843014.1 |
| phosphate transporter [Blastocystis sp. subtype 4] | 337 | 337 | 93% | 6.00E-107 | 40% | XP_014527370.1 |
| putative inorganic phosphate transporter [Emiliania huxleyi CCMP1516] | 330 | 330 | 81% | 2.00E-104 | 46% | XP_005775404.1 |
| sodium/phosphate symporter [Blastocystis sp. ATCC 50177/Nand II] | 330 | 330 | 94% | 3.00E-104 | 39% | OAO13683.1 |
| hypothetical protein AXG93_725s1330 [Marchantia polymorpha subsp. polymorpha] | 329 | 329 | 97% | 7.00E-102 | 38% | OAE31443.1 |
| phosphate transporter [Coccomyxa subellipsoidea C-169] | 324 | 324 | 96% | 1.00E-101 | 38% | XP_005649662.1 |
| hypothetical protein VOLCADRAFT_79832 [Volvox carteri f. nagariensis] | 325 | 325 | 96% | 1.00E-101 | 37% | XP_002947697.1 |
| hypothetical protein AXG93_725s1290 [Marchantia polymorpha subsp. polymorpha] | 325 | 325 | 97% | 3.00E-101 | 38% | OAE31439.1 |
| hypothetical protein AXG93_725s1300 [Marchantia polymorpha subsp. polymorpha] | 325 | 325 | 97% | 2.00E-100 | 38% | OAE31440.1 |
| sodium/phosphate symporter PTB1 [Marchantia polymorpha] | 325 | 325 | 97% | 2.00E-100 | 38% | AQM55164.1 |
| phosphate-repressible phosphate permease [Blastocystis sp. subtype 4] | 319 | 319 | 96% | 5.00E-100 | 38% | XP_014528496.1 |
| phosphate transporter [Blastocystis sp. ATCC 50177/Nand II] | 319 | 319 | 97% | 6.00E-100 | 38% | OAO17103.1 |
| PHO4-domain-containing protein [Coccomyxa subellipsoidea C-169] | 320 | 320 | 97% | 1.00E-99 | 38% | XP_005646297.1 |
| sodium phosphate symporter [Blastocystis sp. subtype 4] | 329 | 329 | 97% | 3.00E-99 | 38% | XP_014525891.1 |
| uncharacterized protein [Blastocystis hominis] | 327 | 327 | 95% | 6.00E-99 | 39% | XP_012894130.1 |
| uncharacterized protein [Blastocystis hominis] | 313 | 313 | 94% | 5.00E-98 | 39% | XP_012894444.1 |
| sodium/phosphate symporter PTB5 [Marchantia polymorpha] | 318 | 318 | 97% | 5.00E-98 | 38% | AQM55168.1 |
| hypothetical protein AXG93_1587s1210 [Marchantia polymorpha subsp. polymorpha] | 317 | 317 | 97% | 6.00E-98 | 36% | OAE34890.1 |
| Phosphate permease PHO89 [Auxenochlorella protothecoides] | 318 | 318 | 100% | 8.00E-98 | 39% | XP_011401900.1 |
| hypothetical protein AXG93_725s1280 [Marchantia polymorpha subsp. polymorpha] | 318 | 318 | 97% | 9.00E-98 | 38% | OAE31438.1 |
| sodium/phosphate symporter PTB8 [Marchantia polymorpha] | 318 | 318 | 97% | 1.00E-97 | 37% | AQM55171.1 |
| hypothetical protein AXG93_725s1340 [Marchantia polymorpha subsp. polymorpha] | 318 | 318 | 97% | 1.00E-97 | 36% | OAE31444.1 |
| sodium/phosphate symporter PTB2 [Marchantia polymorpha] | 317 | 317 | 97% | 3.00E-97 | 36% | AQM55165.1 |
| Phosphate-repressible phosphate permease pho-4 [Zancudomyces culisetae] | 312 | 312 | 96% | 5.00E-97 | 37% | OMH80403.1 |
| hypothetical protein AXG93_725s1310 [Marchantia polymorpha subsp. polymorpha] | 317 | 317 | 97% | 6.00E-97 | 36% | OAE31441.1 |
| hypothetical protein AXG93_523s1240 [Marchantia polymorpha subsp. polymorpha] | 313 | 313 | 97% | 2.00E-96 | 36% | OAE25019.1 |
| sodium/phosphate symporter PTB6 [Marchantia polymorpha] | 314 | 314 | 97% | 5.00E-96 | 37% | AQM55169.1 |
| hypothetical protein KFL_000400430 [Klebsormidium flaccidum] | 312 | 312 | 97% | 2.00E-95 | 37% | GAQ79899.1 |
| hypothetical protein AXG93_1587s1200 [Marchantia polymorpha subsp. polymorpha] | 317 | 317 | 96% | 2.00E-95 | 37% | OAE34889.1 |
| hypothetical protein AXG93_523s1250 [Marchantia polymorpha subsp. polymorpha] | 312 | 312 | 97% | 3.00E-95 | 36% | OAE25020.1 |

**Table S3. BLASTp analysis of the EhV phosphate permease gene against the non-redundant protein database on NCBI.** The top 50 hits using the default BLASTp settings were pulled, and sorted based on the E value in an ascending order.

| **Description** | **Max score** | **Total score** | **Query cover** | **E-**  **value** | **Ident**  **(%)** | **Accession** |
| --- | --- | --- | --- | --- | --- | --- |
| ribonucleoside-diphosphate reductase subunit alpha [Chitinophaga niabensis] | 1085 | 1085 | 98% | 0 | 68% | WP_074242987.1 |
| ribonucleoside-diphosphate reductase subunit alpha [Cnuella takakiae] | 1085 | 1085 | 98% | 0 | 69% | WP_073048483.1 |
| ribonucleoside-diphosphate reductase subunit alpha [Mucilaginibacter sp. 44-25] | 1083 | 1083 | 98% | 0 | 68% | OJW15011.1 |
| ribonucleoside-diphosphate reductase subunit alpha [Solitalea canadensis] | 1081 | 1081 | 98% | 0 | 68% | WP_014679080.1 |
| ribonucleoside-diphosphate alpha subunit [Chrysochromulina sp. CCMP291] | 1078 | 1078 | 98% | 0 | 67% | KOO26551.1 |
| ribonucleoside-diphosphate reductase subunit alpha [Sporocytophaga myxococcoides] | 1076 | 1076 | 98% | 0 | 68% | WP_045468468.1 |
| ribonucleoside-diphosphate reductase subunit alpha [Mucilaginibacter pedocola] | 1075 | 1075 | 98% | 0 | 67% | OOQ58015.1 |
| ribonucleoside-diphosphate reductase subunit alpha [Mucilaginibacter lappiensis] | 1073 | 1073 | 98% | 0 | 67% | WP_076377331.1 |
| ribonucleoside-diphosphate reductase subunit alpha [Filimonas lacunae] | 1072 | 1072 | 98% | 0 | 67% | WP_076379698.1 |
| ribonucleoside-diphosphate reductase alpha chain [Mucilaginibacter sp. OK283] | 1072 | 1072 | 98% | 0 | 67% | SEO65934.1 |
| ribonucleoside-diphosphate reductase alpha chain [Mucilaginibacter sp. OK268] | 1072 | 1072 | 98% | 0 | 67% | SDP05120.1 |
| ribonucleoside-diphosphate reductase subunit alpha [Flavisolibacter sp. LCS9] | 1071 | 1071 | 98% | 0 | 67% | WP_066406697.1 |
| ribonucleoside-diphosphate reductase subunit alpha [Pedobacter sp. BAL39] | 1070 | 1070 | 98% | 0 | 68% | WP_008244459.1 |
| ribonucleoside-diphosphate reductase subunit alpha [Terrimonas ferruginea] | 1070 | 1070 | 98% | 0 | 66% | WP_028786574.1 |
| ribonucleoside-diphosphate reductase alpha chain [Chitinophaga rupis] | 1070 | 1070 | 98% | 0 | 67% | SEM17274.1 |
| ribonucleoside-diphosphate reductase subunit alpha [Chitinophagaceae bacterium PMP191F] | 1070 | 1070 | 98% | 0 | 68% | WP_054281352.1 |
| ribonucleoside-diphosphate reductase subunit alpha [Fabibacter misakiensis] | 1069 | 1069 | 98% | 0 | 67% | WP_069834736.1 |
| ribonucleoside-diphosphate reductase subunit alpha [Pontibacter akesuensis] | 1068 | 1068 | 98% | 0 | 68% | WP_068837657.1 |
| ribonucleoside-diphosphate reductase subunit alpha [Sporocytophaga myxococcoides] | 1068 | 1068 | 98% | 0 | 68% | WP_028979770.1 |
| ribonucleoside-diphosphate reductase subunit alpha, partial [Cytophagales bacterium B6] | 1068 | 1068 | 98% | 0 | 67% | WP_022830657.1 |
| ribonucleoside-diphosphate reductase subunit alpha [Pontibacter indicus] | 1067 | 1067 | 98% | 0 | 68% | WP_076668231.1 |
| ribonucleoside-diphosphate reductase subunit alpha [Mucilaginibacter sp. L294] | 1067 | 1067 | 98% | 0 | 66% | WP_067055453.1 |
| ribonucleoside-diphosphate reductase subunit alpha [Sphingobacteriales bacterium 50-39] | 1066 | 1066 | 98% | 0 | 67% | OJW60196.1 |
| ribonucleoside-diphosphate reductase subunit alpha [Chitinophaga jiangningensis] | 1066 | 1066 | 98% | 0 | 67% | WP_073076957.1 |
| hypothetical protein SCHCODRAFT_75631 [Schizophyllum commune H4-8] | 1066 | 1066 | 97% | 0 | 66% | XP_003034649.1 |
| ribonucleoside-diphosphate reductase alpha chain [Chitinophaga filiformis] | 1066 | 1066 | 98% | 0 | 67% | SDF99156.1 |
| ribonucleoside-diphosphate reductase subunit alpha [Niastella koreensis] | 1065 | 1065 | 98% | 0 | 67% | WP_014221635.1 |
| ribonucleoside-diphosphate reductase subunit alpha [Mucilaginibacter paludis] | 1065 | 1065 | 98% | 0 | 67% | WP_008506930.1 |
| ribonucleoside-diphosphate reductase alpha chain [Niastella yeongjuensis] | 1065 | 1065 | 98% | 0 | 67% | SEN75828.1 |
| ribonucleoside-diphosphate reductase subunit alpha [Sphingobacteriales bacterium 44-61] | 1065 | 1065 | 98% | 0 | 66% | OJW02888.1 |
| ribonucleoside-diphosphate reductase subunit alpha [Pontibacter actiniarum] | 1065 | 1065 | 98% | 0 | 68% | WP_025608564.1 |
| ribonucleoside-diphosphate reductase alpha chain [Pedobacter sp. ok626] | 1064 | 1064 | 98% | 0 | 67% | SDL21009.1 |
| ribonucleoside-diphosphate reductase subunit alpha [Pedobacter antarcticus] | 1063 | 1063 | 98% | 0 | 67% | WP_037440793.1 |
| ribonucleoside-diphosphate reductase subunit alpha [Sphingobacteriales bacterium 46-32] | 1063 | 1063 | 98% | 0 | 66% | OJW32718.1 |
| ribonucleoside-diphosphate reductase alpha chain [Mucilaginibacter mallensis] | 1063 | 1063 | 98% | 0 | 66% | SDT42224.1 |
| ribonucleoside-diphosphate reductase subunit alpha [Pedobacter sp. V48] | 1063 | 1063 | 98% | 0 | 67% | WP_048907701.1 |
| ribonucleoside-diphosphate reductase large chain [Cladophialophora yegresii CBS 114405] | 1063 | 1063 | 98% | 0 | 65% | XP_007758639.1 |
| ribonucleoside-diphosphate reductase alpha chain [Mucilaginibacter gossypiicola] | 1062 | 1062 | 98% | 0 | 66% | SEO21493.1 |
| ribonucleoside-diphosphate reductase subunit alpha [Pedobacter sp. CCM 8644] | 1062 | 1062 | 98% | 0 | 67% | WP_068822521.1 |
| ribonucleoside-diphosphate reductase [Mucilaginibacter sp. PAMC 26640] | 1062 | 1062 | 98% | 0 | 67% | WP_067190639.1 |
| ribonucleoside-diphosphate reductase subunit alpha [Pedobacter sp. PACM 27299] | 1062 | 1062 | 98% | 0 | 67% | WP_062550382.1 |
| ribonucleoside-diphosphate reductase subunit alpha [Roseivirga seohaensis] | 1062 | 1062 | 98% | 0 | 67% | WP_053222508.1 |
| MULTISPECIES: ribonucleoside-diphosphate reductase subunit alpha [Pontibacter] | 1062 | 1062 | 98% | 0 | 68% | WP_040574233.1 |
| ribonucleoside-diphosphate reductase subunit alpha [Belliella pelovolcani] | 1062 | 1062 | 98% | 0 | 67% | WP_076502991.1 |
| ribonucleoside-diphosphate reductase [Pedobacter sp. V48] | 1062 | 1062 | 98% | 0 | 67% | ETZ19946.1 |
| ribonucleoside-diphosphate reductase subunit alpha [Pedobacter heparinus] | 1062 | 1062 | 98% | 0 | 67% | WP_012780859.1 |
| ribonucleoside-diphosphate reductase subunit alpha [Mucilaginibacter sp. OK098] | 1061 | 1061 | 98% | 0 | 67% | WP_073405168.1 |
| ribonucleoside-diphosphate reductase alpha chain [Pontibacter chinhatensis] | 1061 | 1061 | 98% | 0 | 68% | SFG70393.1 |
| ribonucleoside-diphosphate reductase subunit alpha [Solirubrum puertoriconensis] | 1061 | 1061 | 98% | 0 | 67% | WP_059071527.1 |
| ribonucleoside-diphosphate reductase subunit alpha [Pontibacter korlensis] | 1061 | 1061 | 98% | 0 | 68% | WP_046309677.1 |

**Table S4. BLASTp analysis of the EhV ribonucleoside-diphosphate reductase gene against the non-redundant protein database on NCBI.** The top 50 hits using the default BLASTp settings were pulled, and sorted based on the E value in an ascending order.

| **Description** | **Max score** | **Total score** | **Query cover** | **E-**  **value** | **Ident**  **(%)** | **Accession** |
| --- | --- | --- | --- | --- | --- | --- |
| PREDICTED: polyubiquitin-B isoform X2 [Cynoglossus semilaevis] | 152 | 152 | 96% | 1.00E-46 | 95% | XP_008323364.1 |
| Ubiquitin-40S ribosomal protein S27a [Aptenodytes forsteri] | 152 | 152 | 96% | 1.00E-46 | 95% | KFM10709.1 |
| hypothetical protein HELRODRAFT_115575 [Helobdella robusta] | 154 | 154 | 95% | 1.00E-46 | 96% | XP_009028383.1 |
| unnamed protein product [Vitrella brassicaformis CCMP3155] | 155 | 155 | 100% | 1.00E-46 | 91% | CEM07556.1 |
| putative ubiquitin [Corethrella appendiculata] | 151 | 151 | 96% | 2.00E-46 | 95% | JAB58755.1 |
| Ubiquitin-60S ribosomal protein L40 [Zancudomyces culisetae] | 153 | 153 | 98% | 2.00E-46 | 92% | OMH84847.1 |
| Ubiquitin-60S ribosomal protein L40 [Larimichthys crocea] | 153 | 153 | 95% | 2.00E-46 | 96% | KKF28772.1 |
| PREDICTED: ubiquitin-60S ribosomal protein L40-like [Larimichthys crocea] | 152 | 152 | 95% | 2.00E-46 | 96% | XP_010731693.2 |
| PREDICTED: ubiquitin [Papilio polytes] | 151 | 151 | 97% | 2.00E-46 | 94% | XP_013141997.1 |
| PREDICTED: ubiquitin-like [Notothenia coriiceps] | 150 | 150 | 96% | 3.00E-46 | 94% | XP_010769428.1 |
| hypothetical protein M407DRAFT_183161 [Tulasnella calospora MUT 4182] | 150 | 150 | 96% | 3.00E-46 | 95% | KIO16717.1 |
| polyubiquitine protein [Collozoum inerme] | 152 | 202 | 95% | 3.00E-46 | 96% | CAI77901.1 |
| poly-histidine-tagged ubiquitin [Nematocida parisii ERTm1] | 150 | 150 | 98% | 4.00E-46 | 91% | XP_013057853.1 |
| PREDICTED: ubiquitin-like [Rhinolophus sinicus] | 150 | 150 | 97% | 4.00E-46 | 94% | XP_019604376.1 |
| ribosomal protein L40-like isoform 1 [Callorhinchus milii] | 152 | 152 | 97% | 4.00E-46 | 92% | AFM85952.1 |
| ribosomal protein L40-like isoform 1 [Callorhinchus milii] | 152 | 152 | 97% | 5.00E-46 | 92% | AFM86256.1 |
| ubiquitin [Nematocida parisii ERTm1] | 152 | 152 | 95% | 5.00E-46 | 96% | XP_013060057.1 |
| ubiquitin/ribosomal L40 fusion protein [Callorhinchus milii] | 152 | 152 | 97% | 5.00E-46 | 92% | AFM90363.1 |
| PREDICTED: ubiquitin-like [Peromyscus maniculatus bairdii] | 152 | 195 | 95% | 5.00E-46 | 95% | XP_006970728.1 |
| ribosomal protein L40-like isoform 1 [Callorhinchus milii] | 152 | 152 | 97% | 5.00E-46 | 92% | AFM86139.1 |
| ribosomal protein L40-like isoform 1 [Callorhinchus milii] | 152 | 152 | 97% | 6.00E-46 | 92% | AFM86327.1 |
| ribosomal protein L40-like isoform 1 [Callorhinchus milii] | 152 | 152 | 97% | 6.00E-46 | 92% | AFK10626.1 |
| ubiquitin [Chrysochromulina sp. CCMP291] | 151 | 151 | 95% | 6.00E-46 | 95% | KOO29468.1 |
| ribosomal protein L40-like isoform 1 [Callorhinchus milii] | 151 | 151 | 97% | 6.00E-46 | 92% | AFM85613.1 |
| ubiquitin family protein [Ancylostoma duodenale] | 150 | 150 | 97% | 6.00E-46 | 92% | KIH44842.1 |
| ubiquitin A-52 residue ribosomal protein fusion product 1 [Callorhinchus milii] | 151 | 151 | 97% | 6.00E-46 | 92% | NP_001279872.1 |
| ribosomal protein L40-like isoform 1 [Callorhinchus milii] | 151 | 151 | 97% | 6.00E-46 | 92% | AFM86557.1 |
| PREDICTED: ubiquitin-like [Microtus ochrogaster] | 151 | 198 | 95% | 6.00E-46 | 95% | XP_005344417.1 |
| PREDICTED: ubiquitin [Drosophila arizonae] | 150 | 150 | 95% | 6.00E-46 | 95% | XP_017861724.1 |
| ribosomal protein L40-like isoform 1 [Callorhinchus milii] | 151 | 151 | 97% | 6.00E-46 | 92% | AFM86223.1 |
| PREDICTED: ubiquitin [Amphimedon queenslandica] | 151 | 151 | 95% | 6.00E-46 | 95% | XP_011403466.1 |
| Chain B, Solution Structure Of S5a Uim-1UBIQUITIN COMPLEX | 150 | 150 | 95% | 6.00E-46 | 95% | 1YX5_B |
| PREDICTED: ubiquitin [Sinocyclocheilus anshuiensis] | 150 | 150 | 96% | 7.00E-46 | 94% | XP_016332492.1 |
| hypothetical protein AALP_AA8G023100 [Arabis alpina] | 150 | 150 | 97% | 7.00E-46 | 92% | KFK24775.1 |
| GL14086 [Drosophila persimilis] | 150 | 150 | 95% | 7.00E-46 | 95% | XP_002029499.1 |
| GD16200 [Drosophila simulans] | 150 | 150 | 95% | 7.00E-46 | 95% | EDX17243.1 |
| PREDICTED: ubiquitin-like [Aquila chrysaetos canadensis] | 150 | 150 | 95% | 7.00E-46 | 95% | XP_011580094.1 |
| PREDICTED: polyubiquitin-C [Propithecus coquereli] | 150 | 150 | 95% | 7.00E-46 | 95% | XP_012518855.1 |
| Ubiquitin-60S ribosomal protein L40 [Strongyloides ratti] | 151 | 151 | 95% | 7.00E-46 | 95% | CEF70409.1 |
| PREDICTED: ubiquitin-like [Pygocentrus nattereri] | 150 | 150 | 95% | 7.00E-46 | 95% | XP_017546044.1 |
| PREDICTED: ubiquitin [Rhinopithecus roxellana] | 150 | 150 | 95% | 7.00E-46 | 95% | XP_010357545.1 |
| Chain X, Crystal Structure Of K63-Specific Fab Apu.3a8 Bound To K63-Linked Di- Ubiquitin | 150 | 150 | 96% | 7.00E-46 | 94% | 3DVG_X |
| PREDICTED: ubiquitin [Diaphorina citri] | 150 | 150 | 95% | 7.00E-46 | 95% | XP_008485840.2 |
| uncharacterized protein [Babesia microti strain RI] | 149 | 149 | 96% | 7.00E-46 | 94% | XP_012648812.1 |
| ubiquitin-60S ribosomal protein L40 isoform X2 [Tortanus dextrilobatus] | 151 | 151 | 95% | 7.00E-46 | 95% | ALS05233.1 |
| 60S ribosomal protein L40 isoform A [Centropages tenuiremis] | 151 | 151 | 95% | 7.00E-46 | 95% | ALS05064.1 |
| Ubiquitin-40S ribosomal protein S27a [Nipponia nippon] | 150 | 150 | 95% | 7.00E-46 | 95% | KFQ96542.1 |
| hypothetical protein g.14002 [Clastoptera arizonana] | 151 | 151 | 95% | 7.00E-46 | 95% | JAS20459.1 |
| ubiquitin isoform X2 [Microcebus murinus] | 149 | 149 | 95% | 8.00E-46 | 95% | XP_020138558.1 |
| PREDICTED: ubiquitin-60S ribosomal protein L40 [Pygocentrus nattereri] | 151 | 151 | 95% | 8.00E-46 | 95% | XP_017552636.1 |

**Table S5. BLASTp analysis of the EhV DNA polyubiquitin gene against the non-redundant protein database on NCBI.** The top 50 hits using the default BLASTp settings were pulled, and sorted based on the E value in an ascending order.

| **Description** | **Max score** | **Total score** | **Query cover** | **E-**  **value** | **Ident**  **(%)** | **Accession** |
| --- | --- | --- | --- | --- | --- | --- |
| PREDICTED: DNA-directed RNA polymerase II subunit 2-like [Raphanus sativus] | 823 | 823 | 99% | 0 | 41% | XP_018447597.1 |
| RNA polymerase II core subunit [Dictyostelium discoideum AX4] | 821 | 821 | 99% | 0 | 40% | XP_636812.1 |
| PREDICTED: DNA-directed RNA polymerase II subunit RPB2-like [Ipomoea nil] | 816 | 816 | 99% | 0 | 40% | XP_019192937.1 |
| PREDICTED: DNA-directed RNA polymerase II subunit RPB2 [Arachis ipaensis] | 815 | 815 | 99% | 0 | 40% | XP_016179997.1 |
| RNA polymerase II core subunit [Dictyostelium purpureum] | 815 | 815 | 99% | 0 | 40% | XP_003286212.1 |
| hypothetical protein PHAVU_009G230400g [Phaseolus vulgaris] | 813 | 813 | 99% | 0 | 40% | XP_007138707.1 |
| DNA-directed RNA polymerase II subunit RPB2 [Anthurium amnicola] | 812 | 812 | 99% | 0 | 40% | JAT39990.1 |
| hypothetical protein SELMODRAFT_172903 [Selaginella moellendorffii] | 812 | 812 | 99% | 0 | 40% | XP_002972363.1 |
| PREDICTED: DNA-directed RNA polymerase II subunit RPB2-like [Lupinus angustifolius] | 811 | 811 | 99% | 0 | 40% | XP_019419458.1 |
| PREDICTED: DNA-directed RNA polymerase II subunit RPB2 [Amborella trichopoda] | 811 | 811 | 99% | 0 | 40% | XP_006859038.1 |
| hypothetical protein SELMODRAFT_148107 [Selaginella moellendorffii] | 811 | 811 | 99% | 0 | 40% | XP_002971836.1 |
| PREDICTED: DNA-directed RNA polymerase II subunit RPB2 isoform X2 [Oryza brachyantha] | 810 | 810 | 99% | 0 | 40% | XP_015690688.1 |
| hypothetical protein OsJ_11898 [Oryza sativa Japonica Group] | 810 | 810 | 99% | 0 | 40% | EEE59590.1 |
| PREDICTED: DNA-directed RNA polymerase II subunit RPB2 [Lupinus angustifolius] | 810 | 810 | 99% | 0 | 40% | XP_019425799.1 |
| PREDICTED: DNA-directed RNA polymerase II subunit RPB2-like [Sesamum indicum] | 810 | 810 | 99% | 0 | 40% | XP_011100259.1 |
| PREDICTED: DNA-directed RNA polymerase II subunit RPB2 [Oryza sativa Japonica Group] | 810 | 810 | 99% | 0 | 40% | XP_015631661.1 |
| RNA polymerase II second largest subunit [Solanum lycopersicum] | 810 | 810 | 99% | 0 | 39% | NP_001234825.1 |
| PREDICTED: DNA-directed RNA polymerase II subunit RPB2 isoform X1 [Oryza brachyantha] | 810 | 810 | 99% | 0 | 40% | XP_006650345.2 |
| hypothetical protein PHAVU_006G185800g [Phaseolus vulgaris] | 810 | 810 | 99% | 0 | 40% | XP_007148168.1 |
| DNAdirected RNA polymerase, beta subunit [Acanthamoeba castellanii str. Neff] | 810 | 810 | 99% | 0 | 39% | XP_004348530.1 |
| PREDICTED: RNA polymerase II second largest subunit isoform X1 [Solanum lycopersicum] | 809 | 809 | 99% | 0 | 39% | XP_019066807.1 |
| PREDICTED: DNA-directed RNA polymerase II subunit RPB2-like [Capsicum annuum] | 809 | 809 | 99% | 0 | 39% | XP_016541552.1 |
| DNA-directed RNA polymerase II subunit RPB2 [Cajanus cajan] | 809 | 809 | 99% | 0 | 40% | XP_020220417.1 |
| PREDICTED: DNA-directed RNA polymerase II subunit RPB2-like [Vigna radiata var. radiata] | 809 | 809 | 99% | 0 | 40% | XP_014501986.1 |
| PREDICTED: DNA-directed RNA polymerase II subunit RPB2-like [Vigna angularis] | 809 | 809 | 99% | 0 | 40% | XP_017422240.1 |
| DNA-directed RNA polymerase subunit beta [Medicago truncatula] | 809 | 809 | 99% | 0 | 40% | XP_003589372.2 |
| PREDICTED: DNA-directed RNA polymerase II subunit RPB2-like [Solanum tuberosum] | 809 | 809 | 99% | 0 | 39% | XP_006350058.1 |
| RNA polymerase II second largest subunit [Petunia x hybrida] | 809 | 809 | 99% | 0 | 40% | AAY89344.1 |
| PREDICTED: DNA-directed RNA polymerase II subunit RPB2 [Setaria italica] | 808 | 808 | 99% | 0 | 40% | XP_004982316.1 |
| DNA-directed RNA polymerase II subunit RPB2 [Aegilops tauschii subsp. tauschii] | 808 | 808 | 99% | 0 | 40% | XP_020199539.1 |
| PREDICTED: DNA-directed RNA polymerase II subunit RPB2 [Musa acuminata subsp. malaccensis] | 808 | 808 | 99% | 0 | 40% | XP_018684753.1 |
| DNA-directed RNA polymerase II subunit [Dorcoceras hygrometricum] | 808 | 808 | 99% | 0 | 40% | KZV51965.1 |
| PREDICTED: DNA-directed RNA polymerase II subunit RPB2 [Cephus cinctus] | 808 | 808 | 99% | 0 | 39% | XP_015597233.1 |
| PREDICTED: DNA-directed RNA polymerase II subunit RPB2 [Diachasma alloeum] | 808 | 808 | 99% | 0 | 39% | XP_015114488.1 |
| PREDICTED: DNA-directed RNA polymerase II subunit RPB2 [Prunus mume] | 808 | 808 | 99% | 0 | 40% | XP_008225092.1 |
| PREDICTED: DNA-directed RNA polymerase II subunit RPB2 [Cicer arietinum] | 808 | 808 | 99% | 0 | 40% | XP_004499217.1 |
| hypothetical protein PRUPE_ppa000425mg [Prunus persica] | 808 | 808 | 99% | 0 | 40% | XP_007214559.1 |
| RNA polymerase II second largest subunit [Antirrhinum majus] | 808 | 808 | 99% | 0 | 40% | AAY89348.1 |
| PREDICTED: DNA-directed RNA polymerase II subunit RPB2 isoform X1 [Vigna angularis] | 807 | 807 | 99% | 0 | 40% | XP_017436372.1 |
| PREDICTED: DNA-directed RNA polymerase II subunit RPB2 [Vigna radiata var. radiata] | 807 | 807 | 99% | 0 | 40% | XP_014518479.1 |
| PREDICTED: DNA-directed RNA polymerase II subunit RPB2 [Vitis vinifera] | 807 | 807 | 99% | 0 | 40% | XP_002274051.1 |
| PREDICTED: DNA-directed RNA polymerase II subunit 2-like [Brassica napus] | 807 | 807 | 99% | 0 | 40% | XP_013656137.1 |
| PREDICTED: DNA-directed RNA polymerase II subunit RPB2 [Microplitis demolitor] | 806 | 806 | 99% | 0 | 39% | XP_008548228.1 |
| PREDICTED: DNA-directed RNA polymerase II subunit RPB2 [Fopius arisanus] | 806 | 806 | 99% | 0 | 39% | XP_011313723.1 |
| PREDICTED: DNA-directed RNA polymerase II subunit RPB2 [Nelumbo nucifera] | 806 | 806 | 99% | 0 | 40% | XP_010242918.1 |
| PREDICTED: DNA-directed RNA polymerase II subunit RPB2 [Brachypodium distachyon] | 806 | 806 | 99% | 0 | 40% | XP_003561873.1 |
| dna-directed rna polymerase ii subunit rpb2-like protein [Lasius niger] | 806 | 806 | 99% | 0 | 39% | KMQ91453.1 |
| PREDICTED: DNA-directed RNA polymerase II subunit RPB2 [Nasonia vitripennis] | 805 | 805 | 99% | 0 | 39% | XP_008207522.2 |
| PREDICTED: DNA-directed RNA polymerase II subunit RPB2 [Harpegnathos saltator] | 805 | 805 | 99% | 0 | 39% | XP_011137278.1 |
| PREDICTED: DNA-directed RNA polymerase II subunit RPB2 isoform X1 [Nicotiana sylvestris] | 805 | 805 | 99% | 0 | 40% | XP_009765275.1 |

**Table S6 BLASTp analysis of the EhV DNA-directed RNA polymerase subunit B gene against the non-redundant protein database on NCBI.** The top 50 hits using the default BLASTp settings were pulled, and sorted based on the E value in an ascending order.

| **Description** | **Max score** | **Total score** | **Query cover** | **E-**  **value** | **Ident**  **(%)** | **Accession** |
| --- | --- | --- | --- | --- | --- | --- |
| DNA ligase 1-like protein [Chrysochromulina sp. CCMP291] | 639 | 639 | 99% | 0 | 51% | KOO33227.1 |
| predicted protein [Micromonas commoda] | 612 | 612 | 97% | 0 | 51% | XP_002500226.1 |
| PREDICTED: DNA ligase 1 [Nelumbo nucifera] | 607 | 607 | 97% | 0 | 50% | XP_010267243.1 |
| DNA ligase I [Dictyostelium lacteum] | 601 | 601 | 96% | 0 | 48% | KYQ92159.1 |
| DNA ligase I [Dictyostelium fasciculatum] | 600 | 600 | 97% | 0 | 48% | XP_004367109.1 |
| predicted protein [Micromonas pusilla CCMP1545] | 594 | 594 | 97% | 0 | 50% | XP_003057721.1 |
| DNA ligase 1 [Anthurium amnicola] | 593 | 593 | 97% | 0 | 50% | JAT44964.1 |
| DNA ligase [Zostera marina] | 593 | 593 | 97% | 0 | 51% | KMZ64847.1 |
| PREDICTED: DNA ligase 1-like [Musa acuminata subsp. malaccensis] | 593 | 593 | 97% | 0 | 50% | XP_009390651.1 |
| PREDICTED: DNA ligase 1-like [Ziziphus jujuba] | 590 | 590 | 96% | 0 | 49% | XP_015874494.1 |
| ATP-dependent DNA ligase [Gonapodya prolifera JEL478] | 589 | 589 | 97% | 0 | 47% | KXS20737.1 |
| PREDICTED: DNA ligase 1-like [Rhagoletis zephyria] | 588 | 588 | 98% | 0 | 49% | XP_017494953.1 |
| PREDICTED: DNA ligase 1-like [Pyrus x bretschneideri] | 588 | 588 | 96% | 0 | 49% | XP_009335981.1 |
| PREDICTED: DNA ligase 1-like [Solanum tuberosum] | 587 | 587 | 96% | 0 | 50% | XP_006349032.1 |
| PREDICTED: DNA ligase 1 [Cucumis sativus] | 587 | 587 | 97% | 0 | 48% | XP_004145818.1 |
| PREDICTED: DNA ligase 1-like [Brachypodium distachyon] | 587 | 587 | 97% | 0 | 49% | XP_003571920.1 |
| hypothetical protein G7K_2930-t1 [Saitoella complicata NRRL Y-17804] | 587 | 587 | 99% | 0 | 48% | GAO48761.1 |
| DNA ligase [Saitoella complicata NRRL Y-17804] | 586 | 586 | 99% | 0 | 48% | XP_019024697.1 |
| unnamed protein product [Vitis vinifera] | 586 | 586 | 97% | 0 | 50% | CBI17138.3 |
| DNA ligase 1 isoform X2 [Aegilops tauschii subsp. tauschii] | 586 | 586 | 97% | 0 | 49% | XP_020160393.1 |
| DNA ligase I [Morus alba] | 586 | 586 | 97% | 0 | 49% | AJK31582.1 |
| PREDICTED: DNA ligase 1 [Vitis vinifera] | 586 | 586 | 97% | 0 | 50% | XP_002272683.2 |
| PREDICTED: DNA ligase 1 [Halyomorpha halys] | 585 | 585 | 96% | 0 | 48% | XP_014294490.1 |
| DNA ligase 1 [Ananas comosus] | 584 | 584 | 97% | 0 | 50% | XP_020094279.1 |
| PREDICTED: DNA ligase 1 [Solanum lycopersicum] | 584 | 584 | 96% | 0 | 49% | XP_004250982.1 |
| PREDICTED: DNA ligase 1-like [Solanum pennellii] | 584 | 584 | 96% | 0 | 49% | XP_015058981.1 |
| DNA ligase I, ATP-dependent (dnl1) [Spizellomyces punctatus DAOM BR117] | 584 | 584 | 97% | 0 | 49% | XP_016608421.1 |
| hypothetical protein CICLE_v10027871mg [Citrus clementina] | 584 | 584 | 96% | 0 | 49% | XP_006424457.1 |
| ATP-dependent DNA ligase [Mortierella elongata AG-77] | 583 | 583 | 99% | 0 | 48% | OAQ33744.1 |
| PREDICTED: DNA ligase 1-like [Capsicum annuum] | 583 | 583 | 96% | 0 | 49% | XP_016547744.1 |
| PREDICTED: DNA ligase 1 [Nicotiana tomentosiformis] | 583 | 583 | 97% | 0 | 49% | XP_009588837.1 |
| PREDICTED: DNA ligase 1-like [Pyrus x bretschneideri] | 583 | 583 | 96% | 0 | 49% | XP_009345748.1 |
| ATP dependent DNA ligase domain-containing protein [Batrachochytrium dendrobatidis JEL423] | 583 | 583 | 96% | 0 | 47% | OAJ39880.1 |
| PREDICTED: DNA ligase 1-like [Nicotiana tabacum] | 583 | 583 | 97% | 0 | 49% | XP_016450837.1 |
| ligase I [Capsaspora owczarzaki ATCC 30864] | 583 | 583 | 99% | 0 | 47% | XP_004346426.1 |
| PREDICTED: DNA ligase 1 [Oryza brachyantha] | 582 | 582 | 97% | 0 | 49% | XP_015697493.1 |
| PREDICTED: DNA ligase 1-like [Nicotiana sylvestris] | 582 | 582 | 97% | 0 | 49% | XP_009788569.1 |
| PREDICTED: DNA ligase 1-like [Sesamum indicum] | 582 | 582 | 97% | 0 | 49% | XP_011089962.1 |
| PREDICTED: DNA ligase 1-like [Citrus sinensis] | 581 | 581 | 96% | 0 | 49% | XP_006488009.1 |
| PREDICTED: DNA ligase 1-like isoform X1 [Limulus polyphemus] | 580 | 580 | 97% | 0 | 47% | XP_013776199.1 |
| uncharacterized protein [Blastocystis hominis] | 580 | 580 | 97% | 0 | 48% | XP_012898281.1 |
| PREDICTED: DNA ligase 1-like isoform X2 [Limulus polyphemus] | 580 | 580 | 97% | 0 | 47% | XP_013776200.1 |
| PREDICTED: LOW QUALITY PROTEIN: DNA ligase 1-like [Cucumis melo] | 580 | 580 | 97% | 0 | 48% | XP_008465403.1 |
| putative DNA ligase [Oryza sativa Japonica Group] | 580 | 580 | 96% | 0 | 49% | AAL31067.1 |
| PREDICTED: DNA ligase 1 [Oryza sativa Japonica Group] | 580 | 580 | 96% | 0 | 49% | XP_015614473.1 |
| PREDICTED: DNA ligase 1-like [Malus domestica] | 580 | 580 | 96% | 0 | 49% | XP_008359654.1 |
| hypothetical protein BSLG_05068 [Batrachochytrium salamandrivorans] | 579 | 579 | 96% | 0 | 47% | OON04933.1 |
| PREDICTED: DNA ligase 1 [Ipomoea nil] | 579 | 579 | 96% | 0 | 49% | XP_019199515.1 |
| PREDICTED: DNA ligase 1-like [Nicotiana attenuata] | 579 | 579 | 97% | 0 | 49% | XP_019235601.1 |
| DNA ligase 1 [Morus notabilis] | 579 | 579 | 96% | 0 | 49% | XP_010111844.1 |

**Table S7 BLASTp analysis of the EhV DNA ligase gene against the non-redundant protein database on NCBI.** The top 50 hits using the default BLASTp settings were pulled, and sorted based on the E value in an ascending order.

| **Description** | **Max score** | **Total score** | **Query cover** | **E-**  **value** | **Ident**  **(%)** | **Accession** |
| --- | --- | --- | --- | --- | --- | --- |
| cytidine and deoxycytidylate deaminase zinc-binding region [Clostridium sp. KLE 1755] | 205 | 205 | 93% | 6.00E-65 | 63% | WP_021634369.1 |
| cytidine deaminase [Roseburia sp. CAG:197_41_10] | 203 | 203 | 93% | 3.00E-64 | 60% | OLA76780.1 |
| cytidine deaminase [Marvinbryantia formatexigens] | 203 | 203 | 93% | 3.00E-64 | 62% | WP_006863231.1 |
| cytidine deaminase [Eisenbergiella tayi] | 201 | 201 | 93% | 1.00E-63 | 61% | WP_009251923.1 |
| dCMP deaminase [Lachnospiraceae bacterium G11] | 201 | 201 | 91% | 2.00E-63 | 60% | SDA78060.1 |
| cytidine deaminase [Ruminococcus albus] | 200 | 200 | 93% | 4.00E-63 | 59% | WP_013496828.1 |
| deoxycytidylate deaminase [Ruminococcus callidus] | 200 | 200 | 93% | 5.00E-63 | 59% | WP_021681091.1 |
| deoxycytidylate deaminase [Clostridium sp. CAG:299] | 200 | 200 | 95% | 1.00E-62 | 58% | CDD41971.1 |
| cytidine deaminase [Ruminococcus albus] | 199 | 199 | 93% | 1.00E-62 | 59% | WP_074960782.1 |
| cytidine and deoxycytidylate deaminase zinc-binding region [Ruminococcus sp. CAG:330] | 199 | 199 | 93% | 1.00E-62 | 60% | CDE12876.1 |
| cytidine deaminase [Ruminococcus albus] | 199 | 199 | 93% | 1.00E-62 | 60% | WP_002851567.1 |
| deoxycytidylate deaminase [Candidatus Mycoplasma girerdii] | 199 | 199 | 88% | 1.00E-62 | 63% | AIV03437.1 |
| tRNA-specific adenosine deaminase [Fusicatenibacter sp. 2789STDY5834925] | 199 | 199 | 93% | 1.00E-62 | 60% | CUQ37868.1 |
| PREDICTED: deoxycytidylate deaminase isoform X2 [Hippocampus comes] | 200 | 200 | 91% | 1.00E-62 | 59% | XP_019735395.1 |
| dCMP deaminase [Sarcina sp. DSM 11001] | 198 | 198 | 93% | 2.00E-62 | 61% | SDK46516.1 |
| cytidine deaminase [[Clostridium] josui] | 198 | 198 | 91% | 2.00E-62 | 59% | WP_024833975.1 |
| dCMP deaminase [Lachnospiraceae bacterium XBD2001] | 198 | 198 | 93% | 3.00E-62 | 58% | SFT52292.1 |
| cytidine deaminase [Clostridium sp. BNL1100] | 197 | 197 | 91% | 4.00E-62 | 58% | WP_014313144.1 |
| cytidine deaminase [[Clostridium] papyrosolvens] | 197 | 197 | 91% | 4.00E-62 | 58% | WP_020816998.1 |
| cytidine deaminase [[Eubacterium] rectale] | 197 | 197 | 93% | 4.00E-62 | 59% | WP_055224459.1 |
| cytidine deaminase [Clostridium sp. Marseille-P2414] | 197 | 197 | 86% | 4.00E-62 | 63% | WP_066894707.1 |
| Riboflavin biosynthesis protein RibD [Roseburia hominis] | 197 | 197 | 93% | 5.00E-62 | 59% | CUN85505.1 |
| cytidine deaminase [Clostridium sp. M62/1] | 198 | 198 | 93% | 5.00E-62 | 59% | WP_008396409.1 |
| cytidine deaminase [[Eubacterium] rectale] | 197 | 197 | 93% | 5.00E-62 | 59% | WP_012741041.1 |
| deoxycytidylate deaminase [[Eubacterium] rectale] | 197 | 197 | 93% | 6.00E-62 | 59% | WP_015517612.1 |
| deoxycytidylate deaminase [Clostridium sp. CAG:149] | 199 | 199 | 93% | 7.00E-62 | 59% | CCY85577.1 |
| dCMP deaminase [Lachnospiraceae bacterium YSD2013] | 197 | 197 | 89% | 8.00E-62 | 62% | SCX11245.1 |
| cytidine deaminase [Stomatobaculum longum] | 197 | 197 | 93% | 8.00E-62 | 58% | WP_009533138.1 |
| putative uncharacterized protein [Firmicutes bacterium CAG:24] | 197 | 197 | 93% | 8.00E-62 | 58% | CCY22883.1 |
| cytidine deaminase [Lachnospira pectinoschiza] | 197 | 197 | 93% | 9.00E-62 | 60% | WP_074521722.1 |
| MULTISPECIES: cytidine deaminase [Veillonella] | 196 | 196 | 93% | 1.00E-61 | 60% | WP_009354146.1 |
| cytidine deaminase [Ruminococcus albus] | 196 | 196 | 93% | 1.00E-61 | 58% | WP_037288532.1 |
| cytidine deaminase [Roseburia hominis] | 196 | 196 | 93% | 1.00E-61 | 59% | WP_014081241.1 |
| cytidine deaminase [Ruminococcus albus] | 196 | 196 | 93% | 1.00E-61 | 58% | WP_024858214.1 |
| cytidine deaminase [Lachnospira multipara] | 196 | 196 | 93% | 1.00E-61 | 60% | WP_027430996.1 |
| PREDICTED: deoxycytidylate deaminase isoform X1 [Hippocampus comes] | 197 | 197 | 90% | 1.00E-61 | 59% | XP_019735394.1 |
| dCMP deaminase [Lachnospiraceae bacterium C10] | 196 | 196 | 93% | 1.00E-61 | 58% | SCW75575.1 |
| deoxycytidylate deaminase [Fusicatenibacter saccharivorans] | 196 | 196 | 93% | 1.00E-61 | 58% | WP_022462096.1 |
| cytidine deaminase [Butyrivibrio sp. AE2032] | 196 | 196 | 90% | 1.00E-61 | 58% | WP_034449829.1 |
| dCMP deaminase [Lachnospiraceae bacterium KHCPX20] | 196 | 196 | 93% | 1.00E-61 | 58% | SDW91585.1 |
| MULTISPECIES: cytidine deaminase [Clostridiales] | 196 | 196 | 93% | 1.00E-61 | 59% | WP_024739631.1 |
| Deoxycytidylate deaminase [Coprococcus catus GD/7] | 196 | 196 | 91% | 2.00E-61 | 61% | CBK79614.1 |
| tRNA-specific adenosine deaminase [uncultured Roseburia sp.] | 196 | 196 | 93% | 2.00E-61 | 60% | SCI48528.1 |
| cytidine deaminase [Lachnospira pectinoschiza] | 196 | 196 | 93% | 2.00E-61 | 59% | WP_055173815.1 |
| hypothetical protein HMPREF9474_02486 [ [[Clostridium] symbiosum WAL-14163] | 197 | 197 | 93% | 2.00E-61 | 59% | EGA93600.1 |
| dCMP deaminase [Lachnospiraceae bacterium NK3A20] | 196 | 196 | 89% | 2.00E-61 | 61% | SDZ87337.1 |
| dCMP deaminase [Eubacterium sp. CAG:252] | 196 | 196 | 93% | 2.00E-61 | 60% | CDB67579.1 |
| putative deoxycytidylate deaminase [Eubacterium sp. CAG:603] | 196 | 196 | 93% | 2.00E-61 | 61% | CCZ03700.1 |
| hypothetical protein [Lachnospira pectinoschiza] | 196 | 196 | 93% | 2.00E-61 | 59% | WP_022501455.1 |
| putative uncharacterized protein [Eubacterium sp. CAG:86] | 196 | 196 | 93% | 2.00E-61 | 59% | CCX82636.1 |

**Table S8 BLASTp analysis of the EhV deoxycytidylate deaminase gene against the non-redundant protein database on NCBI.** The top 50 hits using the default BLASTp settings were pulled, and sorted based on the E value in an ascending order.

| **Description** | **Max score** | **Total score** | **Query cover** | **E-**  **value** | **Ident**  **(%)** | **Accession** |
| --- | --- | --- | --- | --- | --- | --- |
| hypothetical protein AUJ96_30775 [Armatimonadetes bacterium CG2_30_66_41] | 148 | 148 | 81% | 7.00E-38 | 32% | OIO93211.1 |
| neuraminidase [Sphingobacterium deserti] | 149 | 149 | 81% | 8.00E-38 | 30% | KGE13938.1 |
| hypothetical protein [Sphingobacterium deserti] | 148 | 148 | 81% | 8.00E-38 | 30% | WP_052072353.1 |
| hypothetical protein [Monosiga brevicollis MX1] | 141 | 141 | 77% | 2.00E-35 | 33% | XP_001749157.1 |
| sialidase [Blastopirellula marina] | 133 | 133 | 84% | 5.00E-32 | 29% | WP_002653104.1 |
| Sialidase precursor (Neuraminidase) (NEU1) [uncultured marine group II/III euryarchaeote KM3_149_A03] | 131 | 131 | 81% | 2.00E-31 | 31% | AIF01494.1 |
| Sialidase precursor [Phycisphaerae bacterium SM-Chi-D1] | 131 | 131 | 84% | 4.00E-31 | 28% | AQQ70473.1 |
| hypothetical protein ELVG_00016 [Emiliania huxleyi virus 203] | 120 | 120 | 15% | 2.00E-30 | 93% | AEO98317.1 |
| hypothetical protein AMK72_07160 [Planctomycetes bacterium SM23_25] | 128 | 128 | 78% | 3.00E-30 | 32% | KPK48354.1 |
| hypothetical protein [Arenibacter sp. C-21] | 128 | 128 | 82% | 4.00E-30 | 28% | WP_069859267.1 |
| hypothetical protein [Arenibacter algicola] | 128 | 128 | 82% | 4.00E-30 | 28% | WP_034248627.1 |
| exo-alpha-sialidase [Candidatus Solibacter usitatus] | 127 | 127 | 86% | 8.00E-30 | 29% | WP_011681983.1 |
| exo-alpha-sialidase [Sphingobacteriales bacterium 44-15] | 126 | 126 | 85% | 2.00E-29 | 27% | OJY85620.1 |
| hypothetical protein [Zobellia galactanivorans] | 126 | 126 | 82% | 3.00E-29 | 28% | WP_052725488.1 |
| Sialidase, family GH33 [Zobellia galactanivorans] | 126 | 126 | 82% | 3.00E-29 | 28% | CAZ95099.1 |
| hypothetical protein BGN92_07080 [Sphingobacteriales bacterium 41-5] | 124 | 124 | 81% | 1.00E-28 | 31% | OJU26835.1 |
| exo-alpha-sialidase [Pedobacter heparinus] | 124 | 124 | 80% | 1.00E-28 | 29% | WP_015807528.1 |
| hypothetical protein [Arenibacter palladensis] | 124 | 124 | 89% | 2.00E-28 | 27% | WP_072860600.1 |
| exo-alpha-sialidase [Pedobacter ginsenosidimutans] | 123 | 123 | 89% | 3.00E-28 | 28% | WP_057934357.1 |
| Sialidase precursor [Phycisphaerae bacterium ST-NAGAB-D1] | 125 | 125 | 86% | 4.00E-28 | 28% | AQT67197.1 |
| exo-alpha-sialidase [Pedobacter sp. Leaf194] | 122 | 122 | 83% | 8.00E-28 | 29% | WP_056871625.1 |
| sialidase-1 [Dyadobacter sp. SG02] | 121 | 121 | 86% | 1.00E-27 | 29% | SEI58940.1 |
| sialidase-1 [Kriegella aquimaris] | 120 | 120 | 82% | 2.00E-27 | 28% | SDM70100.1 |
| alpha-sialidase [Streptomyces incarnatus] | 120 | 120 | 79% | 2.00E-27 | 30% | AKJ13297.1 |
| hypothetical protein A2Y12_06210 [Planctomycetes bacterium GWF2_42_9] | 120 | 120 | 90% | 3.00E-27 | 29% | OHB56422.1 |
| glycosyl hydrolase [Cyclobacterium lianum] | 120 | 120 | 89% | 3.00E-27 | 29% | WP_073094119.1 |
| Sialidase (Precursor) [Lentisphaera araneosa] | 119 | 119 | 78% | 6.00E-27 | 27% | WP_007279877.1 |
| hypothetical protein [Haloferula sp. BvORR071] | 119 | 119 | 83% | 8.00E-27 | 29% | WP_075090939.1 |
| sialidase [Alistipes sp. Marseille-P2431] | 119 | 119 | 83% | 8.00E-27 | 29% | WP_064974928.1 |
| alpha-sialidase [Streptomyces durhamensis] | 119 | 119 | 83% | 1.00E-26 | 28% | WP_031158872.1 |
| hypothetical protein AMJ85_06660 [candidate division BRC1 bacterium SM23_51] | 118 | 118 | 81% | 1.00E-26 | 30% | KPL09412.1 |
| hypothetical protein [Phycisphaerae bacterium L21-RPul-D3] | 119 | 119 | 85% | 1.00E-26 | 27% | WP_077539578.1 |
| alpha-sialidase [Streptomyces puniciscabiei] | 118 | 118 | 80% | 3.00E-26 | 30% | WP_055704946.1 |
| alpha-sialidase [Actinobacteria bacterium OK074] | 117 | 117 | 81% | 4.00E-26 | 30% | WP_054217933.1 |
| sialidase [Alistipes timonensis] | 117 | 117 | 83% | 5.00E-26 | 28% | WP_010261374.1 |
| exo-alpha-sialidase [Flectobacillus major] | 117 | 117 | 81% | 6.00E-26 | 27% | WP_026994836.1 |
| exo-alpha-sialidase [Sphingobacteriales bacterium 40-81] | 116 | 116 | 81% | 7.00E-26 | 27% | OJY82384.1 |
| sialidase [Gimesia maris] | 116 | 116 | 87% | 7.00E-26 | 27% | WP_002648532.1 |
| sialidase [Sphingobacterium paucimobilis] | 117 | 117 | 83% | 7.00E-26 | 31% | WP_031301352.1 |
| hypothetical protein M472_19125 [Sphingobacterium paucimobilis HER1398] | 116 | 116 | 83% | 8.00E-26 | 31% | ERJ60870.1 |
| hypothetical protein NIASO_17335 [Niabella soli DSM 19437] | 116 | 116 | 81% | 8.00E-26 | 30% | AHF16456.1 |
| exo-alpha-sialidase [Prevotella sp. DNF00663] | 116 | 116 | 82% | 8.00E-26 | 29% | WP_062435413.1 |
| hypothetical protein [Niabella soli] | 116 | 116 | 81% | 8.00E-26 | 30% | WP_071842297.1 |
| hypothetical protein A2V98_01075 [Planctomycetes bacterium RBG_16_64_12] | 116 | 116 | 81% | 9.00E-26 | 28% | OHB80618.1 |
| sialidase-1 [Algoriphagus alkaliphilus] | 116 | 116 | 80% | 9.00E-26 | 26% | SDA48779.1 |
| hypothetical protein [Prevotella sp. S7 MS 2] | 115 | 115 | 82% | 9.00E-26 | 29% | WP_036899434.1 |
| BNR/Asp-box repeat protein [Prevotella sp. DNF00663] | 115 | 115 | 82% | 1.00E-25 | 29% | KXB78854.1 |
| sialidase-1 [Singulisphaera sp. GP187] | 115 | 115 | 81% | 2.00E-25 | 29% | SIO60382.1 |
| hypothetical protein AW736_25560 [Opitutaceae bacterium TSB47] | 118 | 118 | 79% | 2.00E-25 | 31% | OAM86944.1 |
| sialidase-1 [Dyadobacter soli] | 115 | 115 | 81% | 2.00E-25 | 29% | SDD47000.1 |

**Table S9 BLASTp analysis of the EhV sialidase gene against the non-redundant protein database on NCBI.** The top 50 hits using the default BLASTp settings were pulled, and sorted based on the E value in an ascending order.

| **Description** | **Max score** | **Total score** | **Query cover** | **E-**  **value** | **Ident**  **(%)** | **Accession** |
| --- | --- | --- | --- | --- | --- | --- |
| hypothetical protein USCGTAYLOR_02380 [Chromatiales bacterium USCg_Taylor] | 124 | 124 | 98% | 7.00E-31 | 32% | OOO01415.1 |
| delta 9 acyl-lipid fatty acid desaturase [Niveispirillum irakense] | 120 | 120 | 82% | 2.00E-28 | 33% | WP_051330586.1 |
| acyl-CoA desaturase [Rhodocista sp. MIMtkB3] | 118 | 118 | 82% | 9.00E-28 | 33% | WP_075771662.1 |
| Fatty acid desaturase [Minicystis rosea] | 116 | 116 | 93% | 1.00E-27 | 32% | APR75322.1 |
| hypothetical protein AEM38_01810 [Hyphomonadaceae bacterium UKL13-1] | 116 | 116 | 82% | 5.00E-27 | 31% | AMS30718.1 |
| hypothetical protein [Scytonema sp. HK-05] | 114 | 114 | 96% | 6.00E-27 | 32% | WP_073633180.1 |
| Fatty acid desaturase [Gemmata sp. SH-PL17] | 114 | 114 | 85% | 7.00E-27 | 32% | AMV23314.1 |
| delta 9 acyl-lipid fatty acid desaturase [alpha proteobacterium AAP38] | 114 | 114 | 85% | 3.00E-26 | 33% | WP_054168863.1 |
| Fatty acid desaturase [Minicystis rosea] | 112 | 112 | 87% | 4.00E-26 | 34% | APR85729.1 |
| predicted protein [Thalassiosira pseudonana CCMP1335] | 114 | 114 | 81% | 7.00E-26 | 33% | XP_002286531.1 |
| hypothetical protein BET99_05355 [Marine Group III euryarchaeote CG-Epi2] | 111 | 111 | 94% | 1.00E-25 | 31% | OIR21984.1 |
| delta 9 acyl-lipid fatty acid desaturase [Nitrospirillum amazonense] | 113 | 113 | 93% | 1.00E-25 | 32% | WP_050898015.1 |
| fatty acid desaturase [Nitrospirillum amazonense Y2] | 113 | 113 | 93% | 1.00E-25 | 32% | EGY02116.1 |
| acyl-CoA desaturase [Legionella longbeachae] | 111 | 111 | 93% | 2.00E-25 | 30% | WP_012979245.1 |
| stearoyl-CoA desaturase [Methylotenera mobilis] | 110 | 110 | 87% | 3.00E-25 | 34% | WP_019899024.1 |
| stearoyl-CoA desaturase [Acinetobacter venetianus] | 111 | 111 | 82% | 3.00E-25 | 33% | WP_019384727.1 |
| stearoyl-CoA 9-desaturase [Candidatus Methylopumilus planktonicus] | 110 | 110 | 82% | 3.00E-25 | 34% | WP_046488843.1 |
| uncharacterized protein CMC5_076140 [Chondromyces crocatus] | 111 | 111 | 82% | 3.00E-25 | 34% | AKT43382.1 |
| acyl-CoA desaturase [Acinetobacter venetianus] | 110 | 110 | 82% | 3.00E-25 | 33% | WP_004880338.1 |
| acyl-CoA desaturase [Pajaroellobacter abortibovis] | 110 | 110 | 82% | 4.00E-25 | 33% | WP_075277003.1 |
| MULTISPECIES: acyl-CoA desaturase [Acinetobacter] | 110 | 110 | 82% | 5.00E-25 | 32% | WP_005203763.1 |
| delta 9 acyl-lipid fatty acid desaturase [Solimonas flava] | 111 | 111 | 85% | 5.00E-25 | 32% | WP_028008088.1 |
| stearoyl-CoA 9-desaturase [Gloeocapsa sp. PCC 7428] | 109 | 109 | 82% | 6.00E-25 | 30% | WP_041918521.1 |
| stearoyl-CoA desaturase (delta-9 desaturase) [Acinetobacter sp. CIP 110321] | 110 | 110 | 82% | 7.00E-25 | 32% | WP_016163753.1 |
| Delta-9 acyl-phospholipid desaturase [Gloeocapsa sp. PCC 7428] | 109 | 109 | 82% | 7.00E-25 | 30% | AFZ29742.1 |
| stearoyl-CoA 9-desaturase [Acinetobacter venetianus] | 110 | 110 | 82% | 7.00E-25 | 33% | WP_061517127.1 |
| acyl-CoA desaturase [Chroogloeocystis siderophila] | 108 | 108 | 82% | 8.00E-25 | 29% | WP_073550793.1 |
| stearoyl-CoA desaturase [Acinetobacter sp. COS3] | 109 | 109 | 82% | 9.00E-25 | 32% | WP_023012457.1 |
| stearoyl-CoA 9-desaturase [Acinetobacter venetianus] | 109 | 109 | 82% | 9.00E-25 | 32% | WP_061392723.1 |
| stearoyl-CoA desaturase [Methylophaga thiooxydans] | 108 | 108 | 82% | 1.00E-24 | 33% | WP_008291751.1 |
| acyl-CoA desaturase [Acinetobacter proteolyticus] | 109 | 109 | 82% | 1.00E-24 | 32% | WP_070075348.1 |
| acyl-CoA desaturase [Acinetobacter haemolyticus] | 109 | 109 | 82% | 1.00E-24 | 33% | WP_005082604.1 |
| stearoyl-CoA 9-desaturase [Acinetobacter sp. Ver3] | 108 | 108 | 82% | 2.00E-24 | 31% | WP_035269375.1 |
| acyl-CoA desaturase [Acinetobacter haemolyticus] | 108 | 108 | 82% | 2.00E-24 | 33% | WP_005089999.1 |
| stearoyl-CoA 9-desaturase [Acinetobacter sp. MN12] | 108 | 108 | 82% | 2.00E-24 | 32% | WP_033132835.1 |
| stearoyl-CoA desaturase [Acinetobacter haemolyticus] | 108 | 108 | 82% | 2.00E-24 | 33% | WP_004639521.1 |
| stearoyl-CoA 9-desaturase [Bacteriovorax sp. DB6_IX] | 108 | 108 | 39% | 2.00E-24 | 43% | WP_021279097.1 |
| delta 9 acyl-lipid fatty acid desaturase [Salinisphaera shabanensis] | 110 | 110 | 82% | 2.00E-24 | 33% | WP_021031349.1 |
| MULTISPECIES: stearoyl-CoA desaturase [Acinetobacter] | 108 | 108 | 82% | 2.00E-24 | 33% | WP_008941345.1 |
| stearoyl-CoA desaturase (delta-9 desaturase) [Acinetobacter sp. DSM 11652] | 108 | 108 | 82% | 2.00E-24 | 31% | SEM19175.1 |
| delta 9 acyl-lipid fatty acid desaturase (modular protein) [Candidatus Microthrix parvicella] | 109 | 109 | 95% | 2.00E-24 | 28% | WP_012230591.1 |
| acyl-CoA desaturase [Acinetobacter sp. NIPH 809] | 108 | 108 | 82% | 2.00E-24 | 32% | WP_004653548.1 |
| Fatty acid desaturase [Gemmata sp. SH-PL17] | 108 | 108 | 85% | 2.00E-24 | 30% | AMV26964.1 |
| acyl-CoA desaturase [Candidatus Microthrix parvicella] | 109 | 109 | 95% | 2.00E-24 | 28% | WP_020377025.1 |
| stearoyl-CoA 9-desaturase [Legionella parisiensis] | 108 | 108 | 93% | 2.00E-24 | 29% | WP_058517695.1 |
| stearoyl-CoA 9-desaturase [Acinetobacter haemolyticus] | 108 | 108 | 82% | 3.00E-24 | 32% | WP_061397844.1 |
| acyl-CoA desaturase [Acinetobacter sp. ANC 4105] | 108 | 108 | 82% | 3.00E-24 | 32% | WP_005188354.1 |
| fatty acid desaturase family protein [Legionella longbeachae D-4968] | 106 | 106 | 81% | 4.00E-24 | 30% | EEZ94383.1 |
| hypothetical protein [Calothrix sp. HK-06] | 107 | 107 | 95% | 4.00E-24 | 32% | WP_073618328.1 |
| acyl-CoA desaturase [Acinetobacter sp. ANC 3880] | 108 | 108 | 82% | 4.00E-24 | 31% | WP_005313755.1 |

**Table S10 BLASTp analysis of the EhV fatty acid desaturase gene against the non-redundant protein database on NCBI.** The top 50 hits using the default BLASTp settings were pulled, and sorted based on the E value in an ascending order.
